# Towards robust in vivo quantification of oscillating biomagnetic fields using Rotary Excitation based MRI

**DOI:** 10.1038/s41598-022-19275-5

**Published:** 2022-09-13

**Authors:** Maximilian Gram, P. Albertova, V. Schirmer, M. Blaimer, M. Gamer, M. J. Herrmann, P. Nordbeck, P. M. Jakob

**Affiliations:** 1 Experimental Physics 5, University of Würzburg, Würzburg, Germany; 2 Department of Internal Medicine I, University Hospital Würzburg, Würzburg, Germany; 3 Fraunhofer Institute for Integrated Circuits IIS, Würzburg, Germany; 4 Department of Psychology, University of Würzburg, Würzburg, Germany; 5 Department of Psychiatry, Psychosomatics, and Psychotherapy, Center for Mental Health, University Hospital of Würzburg, Würzburg, Germany

**Keywords:** Biological physics, Imaging techniques, Magnetic resonance imaging, Neurophysiology, Magnetic resonance imaging

## Abstract

Spin-lock based functional magnetic resonance imaging (fMRI) has the potential for direct spatially-resolved detection of neuronal activity and thus may represent an important step for basic research in neuroscience. In this work, the corresponding fundamental effect of Rotary EXcitation (REX) is investigated both in simulations as well as in phantom and in vivo experiments. An empirical law for predicting optimal spin-lock pulse durations for maximum magnetic field sensitivity was found. Experimental conditions were established that allow robust detection of ultra-weak magnetic field oscillations with simultaneous compensation of static field inhomogeneities. Furthermore, this work presents a novel concept for the emulation of brain activity utilizing the built-in MRI gradient system, which allows REX sequences to be validated in vivo under controlled and reproducible conditions. Via transmission of Rotary EXcitation (tREX), we successfully detected magnetic field oscillations in the lower nano-Tesla range in brain tissue. Moreover, tREX paves the way for the quantification of biomagnetic fields.

## Introduction

According to the 2019 Global Burden of Diseases, Injuries, and Risk Factors Study, mental disorders were among the ten most common causes of global burden of diseases, as measured by disability-adjusted life-years^1^. According to analysis of data of previous years, taking into account related suicides, mental disorders could even have a significantly more severe impact^2^. An increase in the prevalence of mental disorders as a result of the influence of the COVID-19 pandemic is considered likely^1^. One of the great goals of current research is to understand the functional principles of the brain, so that those disorders can be studied to understand the underlying mechanisms and to improve their treatment.

In recent years, brain imaging has made great progress. For example, the anatomical structures of the brain are well known from in vivo measurements such as diffusion-weighted magnetic resonance imaging (MRI). Diagnostics currently well-established for functional investigations include techniques that directly measure neuronal activity, such as electroencephalography (EEG) and magnetoencephalography (MEG). Indirect measurement via hemodynamic response is provided by blood oxygenation level dependent (BOLD) functional MRI (fMRI). However, for the precise investigation of functional connectivity and in-depth analysis of brain activity, noninvasive methods with improved spatial and temporal resolution compared to existing methods are required^3^. Consequently, developing a novel method that both meets the requirements of spatiotemporal resolution and direct detection of neuronal activity is of great importance for the progress of brain research. A young branch of MRI research is therefore addressing direct mapping of neuronal activity based on spin-locked MRI techniques^4^.

Direct imaging of neuronal currents occurring during certain activity patterns of the brain was initially attempted by detecting modulations of the relaxation time constant T_2_^*^ or a phase imposition^5^. Since the main magnetic field in clinical scanners is in the range of 1.5…7 T, the system is resonant to oscillating fields in the RF range (radiofrequency, ≈ 1 MHz) and is therefore insensitive to neural oscillations in the Hz range. In a system with a field strength of about ≈ 1 μT, resonant energy absorption of fields with frequencies at the magnitude of Hz is possible^6^. In such low-fields, however, the magnetization M_0_ in thermal equilibrium is very small and thus poor signal-to-noise-ratio (SNR) is to be expected. In 2008, Witzel et al. proposed a completely new approach (SIRS, Stimulus-Induced Rotary Saturation) for detecting tiny oscillating magnetic fields in the Hz range^7^. Instead of using a low-field device, Witzel exploited the rotary saturation effect first described by Redfield in 1955^8^. Within the so-called spin-lock (SL) condition, in the presence of a continuous wave RF pulse rotating at the Larmor frequency, sensitivity to magnetic field fluctuations in the low frequency range can be created^8^ while maintaining high SNR at clinical field strengths^9^. Here, the resonance condition can be shifted by adjusting the SL field strength. In subsequent studies, a modification of the SIRS sequence was proposed, which shows a significantly improved signal scaling behavior and provides sensitivity to the oscillation phase^10,11^. Most importantly, this technique is not based on magnetization saturation, but on excitation of magnetization during spin-lock interaction. This concept, which we refer to hereafter as Rotary EXcitation (REX), holds great potential for the direct detection of neuronal activity^4^, since ultra-weak magnetic oscillations act like pseudo RF pulses in the rotating frame and thus can be detected via sinusoidal signal variations in a positive contrast evaluation (REX weighted contrast). A brief summary of the available studies on rotary saturation and rotary excitation targeting fMRI is provided in the Supplementary Material (S.1).

One of the major challenges in employing the REX technique to detect neuronal activity is its susceptibility to field inhomogeneities^12^. Improvements of basic SL techniques known from T_1ρ_ quantification concerning the compensation of static field inhomogeneities, have not yet been investigated in the context of SIRS or REX experiments. However, compensation of static field inhomogeneities is essential for SL based detection of ultra-weak field oscillations, since static system imperfections are of the same order of magnitude as the dynamic components to be measured^12^. Thus, low susceptibility to inhomogeneities combined with high sensitivity to dynamic magnetic field components is required. Most SIRS^7,11,13–17^ and REX^4,16^ studies were performed without elaborated compensation techniques. For SIRS, a ramped SL technique compensating for ${\mathrm{B}}_{1}^{+}$ imperfections has been introduced^18^, while for REX measurements the widely used composite SL (C-SL) technique^19^ has already been applied. However, the influence of such compensation techniques on the measured REX signals has not been thoroughly investigated so far and furthermore approaches like rotary-echo SL (RE-SL)^20^, balanced SL (B-SL)^21^ and adiabatic excitation^22^, have not been considered. In particular, there is evidence of a strong dependence of REX signals on the SL parameters used (e.g., choice of spin-lock pulse duration t_SL_)^13,23^, which has not yet been considered for compensated REX techniques.

Another major challenge is the experimental validation of new SIRS or REX based methods. Since the interaction of a SL pulse with a weak magnetic field oscillation has to be measured, phantom experiments have been performed so far in which the existing MRI setup had to be equipped with additional hardware. Examples include field-generating loop-coils or dipole electrodes^15^, which need precise calibration and RF shielding within the MRI room. Furthermore, accurate synchronization with the MRI sequence is required. These circumstances slow down technical development and pose a major challenge to reproducibility. In addition, new sequences cannot be tested on humans under in vivo conditions, as this would usually require certification of the developed hardware. Thus, in vivo validations under controlled conditions were not performed and the highly challenging measurements of neuronal biomagnetism have been attempted directly within the fMRI setup^4^.

In this study, the basic REX technique is advanced in three steps. First, we investigate how SL compensation techniques can be used to robustly measure and quantify ultra-weak magnetic field oscillations to achieve direct detection of biomagnetic fields. Thereby, the detected REX signals could potentially be increased while reducing the influence of static field inhomogeneities. The different preparation characteristics of most common compensation techniques were examined by Bloch simulations. Particular attention was paid to the critical parameter of SL duration. Second, we propose a novel method for transmitting the rotary excitation effect onto the object under investigation (tREX, transmitted-Rotary-EXcitation). Using ultra-weak gradient waveforms generated by the built-in MRI gradient system, the proposed method allows to mimic brain activity by transmitting artificial magnetic fields onto the living brain tissue. This not only enables reproducible, rapid validation measurements in phantoms but also a comparison of the compensation techniques under in vivo conditions in humans. Third, we demonstrate how this new technique can pave the way for quantification of biomagnetism, as tREX enables signal calibration in vivo.

## Methods

In the present study, a thorough investigation of the rotary excitation effect for established spin-lock compensation techniques was performed using Bloch simulations, phantom validations and in vivo experiments in human brain tissue. To allow reproduction of our experiments, all measurements were performed using the new tREX concept. Furthermore, the MRI sequences used were implemented in the hardware-independent open-source Pulseq framework^24^ based on Matlab (R2018b, The MathWorks Inc., Natick, Massachusetts, USA). The measurements were carried out on a clinical 3.0 T scanner (MAGNETOM Skyra, Siemens Healthineers, Erlangen, Germany) using a 20-channel head coil and the standard gradient system (max. gradient strength = 45 mT/m, max. slew rate = 200 T/m/s).

### REX preparation modules

Spin-lock preparation modules are commonly classified by their compensation approaches for B_0_ and ${\mathrm{B}}_{1}^{+}$ field inhomogeneities. In this study, the following preparatory sequences were considered:

- standard continuous wave spin-lock (S-SL), no compensation
- rotary-echo spin-lock (RE-SL), ${\mathrm{B}}_{1}^{+}$ compensation^20^
- composite spin-lock (C-SL), single refocused B_0_ and ${\mathrm{B}}_{1}^{+}$ compensation^19^
- balanced spin-lock (B-SL), double refocused B_0_ and ${\mathrm{B}}_{1}^{+}$ compensation^21^

The sequence diagrams of the corresponding preparation modules are shown in the Supplementary Material (Supplementary Fig. 1). All four modules start with a 90° excitation pulse (y′ axis) rotating the magnetization to the x′ axis (notation in rotating frame coordinates). It is known from T_1ρ_ quantification that adiabatic excitation prior to spin-locking is highly beneficial as it allows preventive compensation of ${\mathrm{B}}_{1}^{+}$ imperfections^22^. An identical ${\mathrm{B}}_{1}^{+}$ optimized adiabatic-half-passage pulse (AHP, hyperbolic secant shape, duration 3 ms) was therefore used for all modules. Since the REX method aims at measuring ultra-weak magnetic field oscillations (e.g. $\Delta {\mathrm{B}}_{0}^{\mathrm{stim}}$ = 0 … 100 nT), the compensation of static B_0_ inhomogeneities (e.g. $\Delta {\mathrm{B}}_{0}^{\mathrm{static}}$ = 300 nT for 0.1 ppm at 3 T) during SL interaction is thus of great importance ($\Delta {\mathrm{B}}_{0}^{\mathrm{static}}>\Delta {\mathrm{B}}_{0}^{\mathrm{stim}}$). Among the modules studied, C-SL and B-SL provide compensation with respect to B_0_ inhomogeneities due to their 180° refocusing pulses. Contrary to T_1ρ_ preparation, the final 90° flip-back pulse was omitted in all modules according to Truong's method^4^ for generating REX-weighted contrast.

### Concept of REX imaging

**Figure 1:**
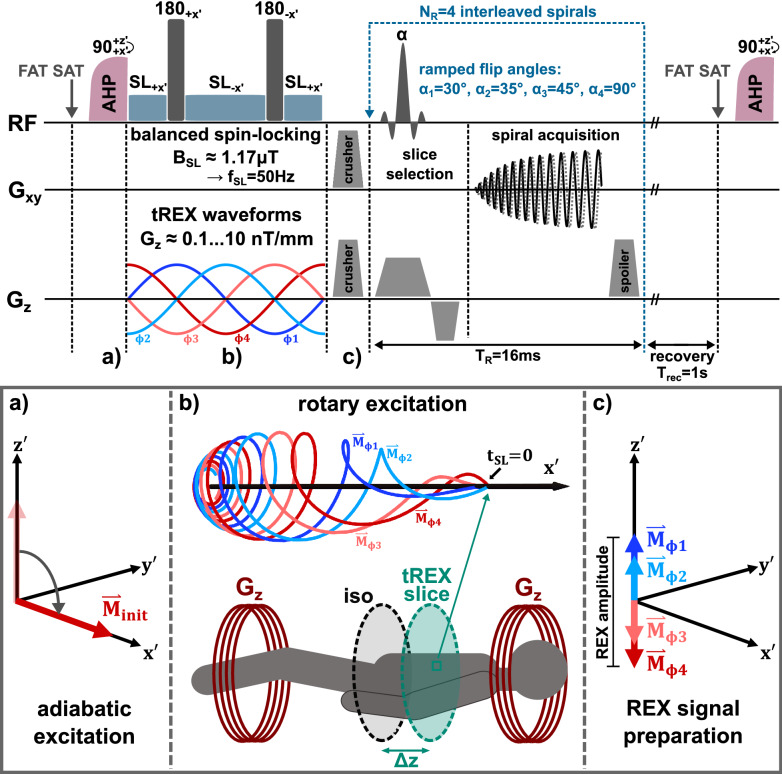
Sequence concept for validation of ultra-weak magnetic field detection using tREX for phantom or in vivo experiments (RF = radio-frequency event, G_x_, G_y_, G_z_ = gradient field event). The upper part of the figure shows the pulse sequence using the B-SL preparation module. In the in vivo experiments, the k-space acquisition was performed with ramped flip angles and four interleaved spirals were used for a fast readout of the REX weighted contrast. In the phantom experiments, the spiral readouts were replaced by TSE readouts. Subfigures (**a**–**c)** explain the individual steps of magnetization behavior. After fat saturation, an adiabatic excitation pulse flips the magnetization to the transverse plane as shown in (**a**). This is followed by one of the four spin-lock preparation modules (B-SL is illustrated exemplarily). During SL preparation, the interaction with the ultra-weak magnetic field oscillation takes place. In the tREX approach the oscillating field is generated by sinusoidal waveforms of the built-in gradient system. To obtain a non-zero amplitude, the measurement must be performed in an offcenter slice, as shown in (**b**). The excitation process of the magnetization perpendicular to the spin-lock axis is dependent on the relative phase between spin-lock pulse and tREX waveform. At the beginning of the spin-lock pulse (t_SL_ = 0) the magnetization is aligned along the x′-axis. The perpendicular component increases due to rotary excitation effect and decays simultaneously with the relaxation time constants T_1ρ_ and T_2ρ_. After the preparation module, remaining transverse magnetization is crushed. The resulting longitudinal magnetization in (**c**) depends on the relative phase according to the trajectories shown in (**b**).

The full sequence design implemented in Pulseq is shown in Fig. 1. Following the REX preparation, strong crusher gradients were applied to dephase remaining transverse magnetization. Thus, only the z-component of the magnetization ${\vec{\mathrm{M}}}_{\mathrm{REX}}$ present at the end of SL interaction was measured. In the resonance case ${\mathrm{f}}_{\mathrm{SL}}={\mathrm{f}}_{\mathrm{stim}}$, the functional relationship between the z-component and the relative phase ϕ between the SL pulse and the stimulus (magnetic field oscillation) is described by a sine function^4,12^. Without further assumptions or simplifications, the sinusoidal signal ${\mathrm{S}}_{\mathrm{REX}}\left(\vec{\mathrm{r}},\upphi \right)$ can be generally described as follows:

1 $${\mathrm{S}}_{\mathrm{REX}}\left(\vec{\mathrm{r}},\upphi \right)=\mathrm{a}\left(\vec{\mathrm{r}}\right)\cdot \mathrm{sin}\left[\upphi \cdot \mathrm{m}\left(\vec{\mathrm{r}}\right)+{\mathrm{\varphi }}_{0}\left(\vec{\mathrm{r}}\right)\right]\cdot \sqrt{2}+\mathrm{b}\left(\vec{\mathrm{r}}\right)$$

Since, off-resonance effects cannot be completely avoided during REX preparation or imaging, the measured signal might preserve an offset $\mathrm{b}\left(\vec{\mathrm{r}}\right)$ and can manifest a phase shift ${\mathrm{\varphi }}_{0}\left(\vec{\mathrm{r}}\right)$ with respect to ϕ^12^. The sinusoidal signal is 2π-periodic only in the exact resonance case and therefore a modulation factor $\mathrm{m}\left(\vec{\mathrm{r}}\right)$ was introduced. The amplitude $\mathrm{a}\left(\vec{\mathrm{r}}\right)$ is primarily influenced by the strength of the oscillating field and is used as the measure of magnetic stimulus detection. This can be determined either by signal fitting with the sinusoidal function (Eq. 1), or by calculating the standard deviation of the signal for N different ϕ^4^:

2 $${\mathrm{A}}_{\mathrm{REX}}\left(\vec{\mathrm{r}}\right)=\left|\mathrm{a}\left(\vec{\mathrm{r}}\right)\right|=\sqrt{\frac{1}{\mathrm{N}-1} \sum_{\mathbf{n}=1}^{\mathbf{N}}{\left[{\mathrm{S}}_{\mathrm{REX}}\left(\vec{\mathrm{r}},{\upphi }_{\mathrm{n}}\right)-{\overline{\mathrm{S}} }_{\mathrm{REX}}\left(\vec{\mathrm{r}}\right)\right]}^{2}}$$

A complete REX experiment thus consists of the acquisition of typically N = 5…20 REX weighted images with different phases ϕ and the evaluation of the amplitude A_REX_ as an indicator for neuronal activity according to Eq. (2) in pixel-wise evaluations. In order to scale the REX amplitude, the magnitude was normalized with the reference signal S_0_ of the imaging sequence without preparation. Simulation results were normalized accordingly with M_0_ = 1.

### Concept of tREX experiments

An essential part of the present study is the realization and validation of REX experiments without using external hardware such as loop-coils or dipole-electrodes for field generation^15^. Instead, we propose a new approach to study the interaction of ultra-weak magnetic field oscillations with spin-locking fields under in vivo conditions using exclusively the standardized hardware on a clinical MRI system. For this purpose, the built-in gradient system was utilized. The gradients, which usually cause spin dephasing for the aim of spatial encoding, were controlled to generate small magnetic oscillations in selected offcenter slices. Thus, the effect of rotary excitation was transmitted (tREX) in the field of view (FOV) within an offcenter slice in distance $\mathrm{\Delta z}$ to the magnets isocenter (Fig. 1b). Magnetic field oscillations with amplitudes in the range 1 … 100 nT at a frequency of f_stim_ = 50 Hz were aspired in $\Delta z$ = 10 mm offcenter slices. The gradient waveform ${\mathrm{G}}_{\mathrm{z}}(\mathrm{t})$ was calculated as follows:

3 $${\mathrm{G}}_{\mathrm{z}}(\mathrm{t})=\frac{\Delta {\mathrm{B}}_{0}^{\mathrm{stim}}}{\mathrm{\Delta z}}\cdot \mathrm{sin}\left[2\uppi\, {\mathrm{f}}_{\mathrm{stim}}\cdot \mathrm{t}+\upphi \right]$$

The tREX concept can also be carried out with the x- or y-gradient or with a superposition of all three gradients in an oblique slice. Here, tREX benefits from the very precise control of gradient coils and enables the full implementation of REX experiments within the Pulseq framework.

### Bloch simulation of REX modules

The four REX modules were investigated with regard to their different preparation characteristics with respect to B_0_ and ${\mathrm{B}}_{1}^{+}$ inhomogeneities using numerical Bloch simulations. The work of Ueda et al. on the dynamics of magnetization under stimulus-induced rotary saturation serves as an important reference^25^. The basic concepts can be adopted for REX. In contrast to Ueda^25^, the Bloch equations, which are described for the REX case by a system of nonlinear differential equations, were solved numerically. A Runge–Kutta method (4th order, RK4) implemented in Matlab was used for this purpose. Details of the simulation can be found in the Supplementary Material (S.3).

The first simulation investigates the influence of the spin-lock parameters t_SL_ and f_SL_ on the amplitude A_REX_. When examining the behavior regarding f_SL_, a resonance peak at f_SL_ = f_stim_ is expected according to^4,25^. In the second simulation, the t_SL_ characteristics for variable f_stim_ was investigated. In further simulations presented in the Supplementary Material, it was tested whether the relaxation times T_1ρ_ and T_2ρ_ or the field strength of the stimulus $\Delta {B}_{0}^{\mathrm{stim}}$ have an influence on the t_SL_ characteristics.

In a final simulation, the performance of the REX amplitude in the presence of B_0_ field imperfections was investigated. For this purpose, a static magnetic field inhomogeneity component $\Delta {\upomega }_{0}^{\mathrm{static}}=2\mathrm{\pi \Delta }{\mathrm{f}}_{0}^{\mathrm{static}}=\upgamma {\mathrm{\Delta B}}_{0}^{\mathrm{static}}$ was assumed (Eq. 5–6, Supplementary Material S.3).

### Phantom experiments

For the phantom experiments, a gel phantom was constructed on agar–agar basis. The Agarose (Agar Kobe I, A2113, A. Hartenstein GmbH) was dissolved in demineralized water at a concentration c = 0.75% and doped with 0.25 mmol/L Gd-DTPA (Gadopentetat-Dimeglumin, Magnograf). The gel was prepared in a cylindrical sample tube (Ø = 9 cm, h = 16 cm, ≈ 1 L).

In initial preparatory experiments, it was investigated whether the ultra-weak gradient waveforms required for the tREX method can be generated with the standard built-in gradient system. The target was the transmission of magnetic field oscillations with $\Delta {B}_{0}^{stim}$ = 1 … 100 nT, e.g. in a 10 mm offcenter slice. For this purpose, trajectory measurements were performed according to^26^ in the Agarose phantom. Sinusoidal gradient waveforms (Eq. 3) with a frequency f_stim_ = 50 Hz and amplitudes in the range 0.02 … 20 nT/mm (15 logarithmically spaced steps) were generated. The relative phase of the oscillating gradients was varied in the range 0 … 2π (10 linearly spaced steps). For the measurement of the gradient trajectories realized by the setup, the phase evolution was recorded in a thin offcenter slice (2 mm thickness) at a distance of 25 mm from the isocenter for t_acq_ = 30 ms (50 kHz bandwidth). To correct for static off-resonance effects, the observed phase evolution was rectified by a reference scan (tREX gradients switched off). The gradient trajectory was subsequently determined from the discrete derivative of the phase evolution. The results of the trajectory measurements were compared with the set gradient stimuli.

For the verification of the rotary excitation effect transmitted by gradient oscillations, a series of experiments was performed in an axial tREX slice (thickness 5 mm, distance from the isocenter 10 mm) in the Agarose phantom with $\Delta {B}_{0}^{stim}$ = 50 nT. REX weighted images with 30 relative phases ϕ = 0 … 3π were acquired. For the pixel-wise determination of REX amplitude maps, both the standard deviation of the measured signal and a sinusoidal fit were evaluated in the range 0 … 2π for the first 20 phases. Control measurements were performed with the tREX gradients switched off (stimulus off). Additionally, the characteristic of the measured REX amplitude for different t_SL_ was investigated and compared with the results of the Bloch simulation. Finally, the t_SL_ characteristic was investigated in a ROI (region-of-interest) based evaluation. For imaging of the REX signals, a turbo spin echo (TSE) readout with centric encoding was used in all phantom experiments (T_E_ = 12 ms). Due to the phase encoding, REX experiments with identical relative phases had to be repeated several times. The FOV was 150 × 150 mm^2^ and the matrix 96 × 96. The turbo factor of the TSE readout was 4, so 96/4 = 24 identical REX preparations were performed for the acquisition of a single REX weighted image. A delay T_rec_ = 1 s was used between the individual preparations with TSE readouts for longitudinal magnetization recovery.

In the present study, the B_0_ field homogeneity was adjusted in the tREX slice by an extended 2nd order shimming routine prior to the actual field detection experiments. ${B}_{1}^{+}$ and B_0_ mapping was performed in the same tREX slice with identical FOV settings for control. Thus, the spatially dependent off-resonances ${\mathrm{\Delta f}}_{0}^{\mathrm{static}}(\vec{\mathrm{r}})$ were determined in the measured FOV. In order to study REX amplitude behavior under the influence of well-defined B_0_ field inhomogeneities, an additional constant gradient was applied in x direction during the SL interaction. The gradient amplitude was varied in different experiments in the range 0 … 32 Hz/cm (5 steps), corresponding to a maximum gradient strength of ≈ 75 nT/mm.

### In vivo measurements

All in vivo experiments were performed in brain tissue of a healthy volunteer. The participant was screened for contraindications to MRI and written informed consent was obtained. The study complied with the Helsinki Declaration on Human Experimentation and was approved by the local institutional ethics board (Ethics Committee of the Institute for Psychology of the Faculty for Human Sciences, University of Würzburg). For the tREX experiment, a slice (thickness 5 mm, distance from the isocenter 10 mm) was chosen that partly intersects the frontal sinus, which exhibits increased field inhomogeneities^27^, in the frontal lobe. B_0_ and ${\mathrm{B}}_{1}^{+}$ mapping was carried out for control. Thus, the REX effect was observed in vivo under controlled conditions and the interference of natural field inhomogeneities with the detection of magnetic field oscillations in brain tissue could be investigated in detail.

For fast in vivo imaging of the REX effect, spiral acquisitions were carried out. In order to reduce the influence of T_2_* relaxation and off-resonance effects, short readout durations (t_adc_ = 11 ms, T_R_ = 16 ms) were used and the k-space was sampled in four segments using interleaved spirals and ramped flip angles (α_k_ = 30°, 35°, 45°, 90°) for slice excitation (Fig. 1). This succession of flip angles produces an approximately uniform transverse magnetization in each interleave, while maximizing the measurement signal. A similar approach has already been used for T_1ρ_ quantification^28^. Hereby, an approximate pure REX weighted contrast can be generated in the final images. For longitudinal magnetization recovery, a delay T_rec_ = 1 s was inserted between each individual REX scan.

The tREX method was investigated in vivo in a comparative study considering best and worst case choice of SL pulse durations. The results of the Bloch simulation regarding t_SL_ characteristics (Eq. 6) were taken into account for the choice of t_SL_. In the best case measurements, the following t_SL_ values were used: 77.5 ms (S-SL), 70 ms (RE-SL), 80 ms (C-SL), 80 ms (B-SL). For the worst case measurements, the following values were used: 82.5 ms (S-SL), 80 ms (RE-SL), 70 ms (C-SL), 60 ms (B-SL). For each measurement, pixel-wise REX amplitude maps were calculated using the standard deviation (Eq. 2). In addition, for the best case measurements, amplitude maps were computed from the complex signal fit (Eq. 1).

Finally, it was examined whether the tREX method can be utilized as a calibration technique for the quantification of magnetic field oscillations. Measurements based on the B-SL preparation module were repeated for different stimulus field strengths. The measured amplitudes A_REX_ were tested for correlation with $\Delta {\mathrm{B}}_{0}^{\mathrm{stim}}$ in a ROI-based evaluation and compared with the control amplitude (stimulus off). Linear regression was performed to test whether tREX is suitable for spatially dependent calibration of REX amplitudes and thus can be used for in vivo quantification of neuronal activity.

### Ethics approval and consent to participate

All experimental procedures were in accordance with institutional guidelines and were approved by local authorities.

## Results

### Bloch Simulation: prediction of A_REX_ maxima

**Figure 2:**
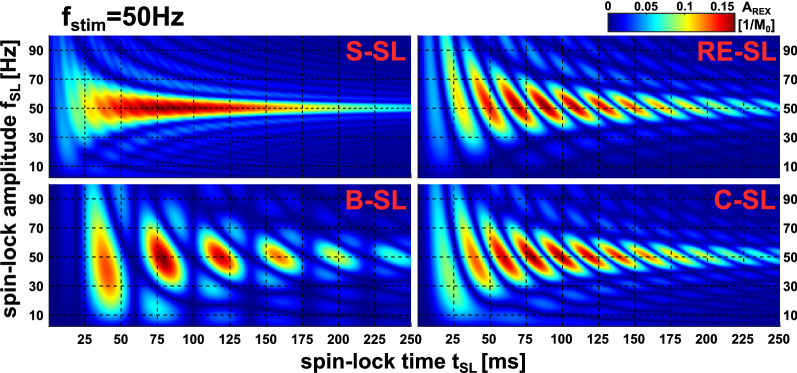
Simulation of the A_REX_ characteristic for different spin-lock preparation modules with f_stim_ = 50 Hz at various spin-lock frequencies f_SL_ and various spin-lock pulse durations t_SL_. Here, f_SL_ was varied from 2.5…100 Hz in 0.5 Hz steps and t_SL_ was varied from 0.5…250 ms in 0.5 ms steps. $\Delta {\mathrm{B}}_{0}^{\mathrm{stim}}$ = 50 nT was assumed. From the Bloch simulations, different module characteristics regarding their resonance behavior and optimal spin-lock pulse durations can be obtained. In the variation of f_SL_ the expected resonance behavior is evident. For short pulse durations (< 25 ms), rotary excitation is hardly observed. For increasing pulse durations, the influence of relaxation processes increases, so that a signal drop is noticeable. While the S-SL module for the resonance condition f_stim_ = f_SL_ = 50 Hz shows a behavior essentially independent of the pulse duration, one recognizes a complex structure for the compensated modules. For certain spin-lock durations, the A_REX_ sensitivity is maximal, while for identical frequencies and different t_SL_, it is minimized.

Figure 2 shows the results of the t_SL_/f_SL_ characteristics as heat maps for the different preparation modules. For all modules, as expected, the highest REX amplitudes were observed at f_SL_ = f_stim_ = 50 Hz in the resonance condition. However, the behavior of A_REX_ when varying t_SL_ is more complex. In the resonance case, alterations of A_REX_ can be observed. These are particularly distinct for the modules equipped with compensation techniques, e.g. with B-SL local maxima occur at t_SL_ ≈ 40, 80, 120, 160 ms, whereas minima (A_REX_ ≈ 0) occur at t_SL_ ≈ 60, 100, 140, 180 ms. Thus, the periodic intervals Δt_SL_ of A_REX_ maxima are: 9.8 ms for S-SL, 19.9 ms for RE-SL, 20.0 ms for C-SL, 40 ms for B-SL. The effect of minima/maxima formation was visually illustrated in the Supplementary Material (Supplementary Fig. 2).

**Figure 3:**
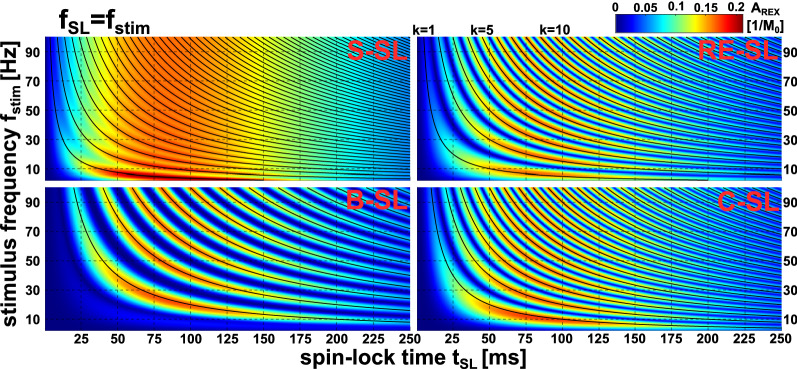
Simulation of A_REX_ sensitivity for different spin-lock preparation modules at various frequencies in the resonant case (f_stim_ = f_SL_) and various spin-lock pulse durations t_SL_. Depending on the resonance frequency, a different spin-lock pulse duration is suitable for maximizing A_REX_. The stimulus frequency f_stim_ was varied from 2.5…100 Hz in 0.5 Hz steps and the REX resonance condition f_SL_ = f_stim_ was considered. $\Delta {\mathrm{B}}_{0}^{\mathrm{stim}}$ = 50 nT was assumed and t_SL_ was varied from 0.5…250 ms in 0.5 ms steps. The black line shows the k-th maximum calculated by the empirically derived prediction law (Eq. 4). For the S-SL module, the maxima are less sharply defined than in the case of the compensated modules. The results show that a parameter choice matched to the stimulus frequency is of high importance for maximizing A_REX_. These results were used for a detailed parameter optimization and to build the basic framework for the t_SL_ choice in the phantom and in vivo measurements carried out in this work.

In Fig. 3, the oscillation phenomenon can be examined for variable f_stim_. Here it can be seen that the periods are shortest for S-SL and doubled for RE-SL and C-SL. The period of B-SL is quadrupled compared to S-SL. The length of the periods depends on f_stim_ and a module dependent factor η. The period length can be described by $\Delta {\mathrm{t}}_{\mathrm{SL}}=\frac{\upeta }{2 {\mathrm{f}}_{\mathrm{stim}}}$. For S-SL and RE-SL the positions of the maxima are shifted. The following approximation was empirically derived for the prediction of the k-th A_REX_ maximum:

4 $${\mathrm{t}}_{\mathrm{SL}}\left(\mathrm{k}\right)=\frac{\upeta }{2 {\mathrm{f}}_{\mathrm{stim}}}\cdot (\mathrm{k}-\upkappa )$$

with $$\mathrm{k}\in {\mathbb{N}}$$,

$\upeta$ = 1 for S-SL, $\upeta$ = 2 for RE-SL, $\upeta$ = 2 for C-SL, $\upeta$ = 4 for B-SL, $\upkappa$ = 1/4 for S-SL, $\upkappa$ = 1/2 for RE-SL, $\upkappa$ = 0 for C-SL, $\upkappa$ = 0 for B-SL.

This rule applies for the modules RE-SL, C-SL and B-SL with a mean deviation of − 0.05% in the simulated range. For S-SL, where minima and maxima are not distinct, the prediction is less accurate (− 4.6%). The results concerning the influence of the T_1ρ_ and T_2ρ_ relaxation times on the position of the maxima are shown in the Supplementary Material (Supplementary Fig. 3). Here, only minor deviations were found with regard to the positions of the maxima. Essentially, short relaxation times hamper the measurement at high SL times. The magnitude of the stimulus $\Delta {\mathrm{B}}_{0}^{\mathrm{stim}}$ determines the magnitude of the observed amplitude A_REX_, with a clear linear correlation (R^2^ = 0.998, Supplementary Fig. 4). No significant influence on t_SL_ characteristic was found. Thus, Eq. (4) describes the occurrence of A_REX_ maxima in a general way. Therefore, for f_stim_ = 50 Hz, the following SL times were used in further simulations and measurements: 77.5 ms for S-SL, 70 ms for RE-SL, 80 ms for C-SL, 80 ms for B-SL.

### Phantom measurements

The validation of ultra-weak gradient waveforms is reported in the Supplementary Material (Supplementary Fig. 5). The measurements confirm that the built-in gradient system enables magnetic field oscillations to be generated precisely with amplitudes 1 … 20 nT/mm (R^2^ > 0.99). In this range, both amplitude and phase could be set precisely. The mean deviation of the adjusted amplitude was − 1.16%. A waveform with 0.1 nT/mm represents the limit. Here, a slight oscillation with high noise level could still be observed in the trajectory measurement. However, R^2^ = 0.67 was found this case. Below this limit, no oscillation of the tREX gradient could be detected. Thus, tREX experiments up to 1 nT can be performed within a 10 mm offcenter slice.

**Figure 4:**
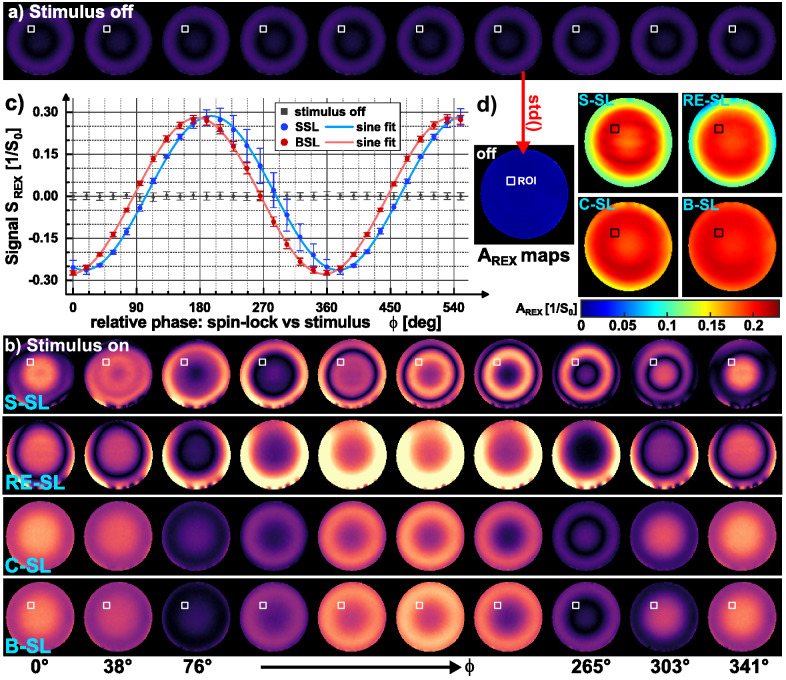
Phantom measurements for feasibility demonstration of the tREX method. (**a**) The reference scans (stimulus off) and (**b**) shows the REX weighted magnitude images (S_REX_) for 10 different relative phases between spin-lock pulse and transmitted oscillation. In (**c**), the signal intensity, which is used for A_REX_ calculation, was plotted against the relative phase in a ROI based evaluation exemplary for S-SL and B-SL. In addition, the reference scan results were plotted. The standard deviation calculated pixel-by-pixel from the phase variation is shown in the A_REX_ maps in (**d**). The measurements performed with B-SL show reduced banding artifacts in the S_REX_ images, which is due to the improved compensation of field inhomogeneities. Thus, the A_REX_ map is most homogenous as well. Furthermore, decreasing A_REX_ at the edge of the phantom is visible for S-SL and RE-SL. This correlates with deviations of the ${\mathrm{B}}_{1}^{+}$ field (Supplementary Fig. 7). The measurements were performed in an axial tREX slice (Δz = 10 mm, 5 mm thickness). Other sequence parameters were: f_SL_ = f_stim_ = 50 Hz, $\Delta {\mathrm{B}}_{0}^{\mathrm{stim}}$ = 50 nT, FOV = 150 × 150 mm^2^, matrix = 96 × 96.

Figure 4 presents the proof-of-concept of the tREX method. The expected sinusoidal signal for different phases ϕ could be clearly observed (R^2^ = 0.999). However, a strong formation of banding artifacts is evident in the REX-weighted images for S-SL, which was significantly reduced for B-SL. All measurements were performed in the expected A_REX_ maximum (according to Bloch simulations). Yet, differences in the calculated A_REX_ maps are evident. B-SL (${\mathrm{A}}_{\mathrm{REX}}^{\mathrm{BSL}}$=0.1994 ± 0.0089) shows a more homogeneous and on average higher REX amplitude than S-SL (${\mathrm{A}}_{\mathrm{REX}}^{\mathrm{SSL}}$ = 0.173 ± 0.033). The REX amplitudes of RE-SL and C-SL were ${\mathrm{A}}_{\mathrm{REX}}^{\mathrm{RESL}}$ = 0.165 ± 0.037 and ${\mathrm{A}}_{\mathrm{REX}}^{\mathrm{CSL}}$ = 0.193 ± 0.017. The control measurement (stimulus off using B-SL) exhibited no significant oscillation (${\mathrm{A}}_{\mathrm{REX}}^{\mathrm{off}}$ = 0.0060 ± 0.0038). From the pre-calibration scans, the following B_0_ and ${\mathrm{B}}_{1}^{+}$ field deviations were observed within the tREX slice: Δf_0_ = − 2.3 ± 3.5 Hz and ${\mathrm{B}}_{1}^{+}$ = 96.4 ± 6.8%. Here, ${\mathrm{B}}_{1}^{+}$ represents the ratio of the measured to the nominal ${\mathrm{B}}_{1}^{+}$ field strength. In the Supplementary Material, the corresponding maps $\mathrm{a}\left(\vec{\mathrm{r}}\right)$, $\mathrm{b}\left(\vec{\mathrm{r}}\right)$, ${\mathrm{\varphi }}_{0}\left(\vec{\mathrm{r}}\right)$ and $\mathrm{m}\left(\vec{\mathrm{r}}\right)$ fitted with Eq. (1) were compared and the respective ΔB_0_ and ${\mathrm{B}}_{1}^{+}$ field maps are attached (Supplementary Figs. 6–7).

**Figure 5:**
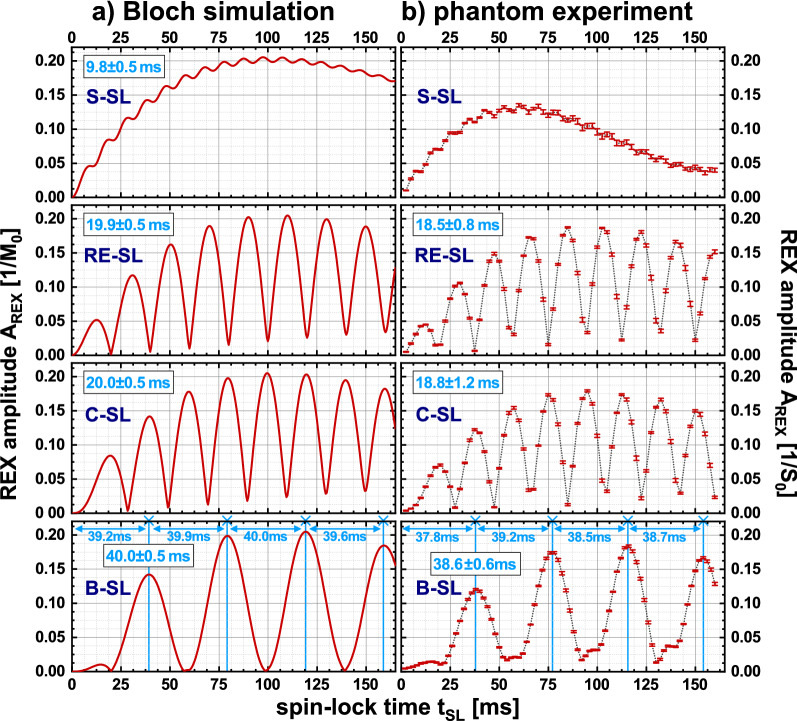
Experimental verification of Bloch simulation t_SL_ characteristics in the phantom experiment. In (**a**), the results of the Bloch simulation are plotted. (**b**) The A_REX_ amplitudes experimentally determined by a t_SL_ variation measured in the Agarose phantom. Here, t_SL_ was varied from 0…160 ms for each preparation module. To achieve feasible measurement times, six relative phases ϕ were varied per amplitude calculation. The respective Δt_SL_ periods are noted in blue. Due to the low amplitude of the oscillation, no period duration was determined for the phantom results of the S-SL module. The qualitative behavior of A_REX_ maxima could be observed in the phantom measurements in good agreement with the simulation results. The period lengths of the measurements are slightly smaller than in the simulation, which could be due to a deviation of the actual spin-lock amplitude (${\mathrm{B}}_{1}^{+}$ inhomogeneity). In the simulation and experiment, the periods are shortest for S-SL and doubled for RE-SL and C-SL. The period of B-SL is quadrupled.

Figure 5 depicts comparisons between the measured t_SL_ characteristics and the Bloch simulation results for the resonance condition. The data show high agreement, confirming the existence of t_SL_ minima and maxima. The following Δt_SL_ periods were obtained in good agreement with the simulation results: 18.5 ± 0.8 ms for RE-SL, 18.8 ± 1.2 ms for C-SL, 38.6 ± 0.6 ms for B-SL. The shift of RE-SL vs C-SL was 9.3 ± 0.4 ms. No values were determined for S-SL due to the weak oscillation strength.

**Figure 6:**
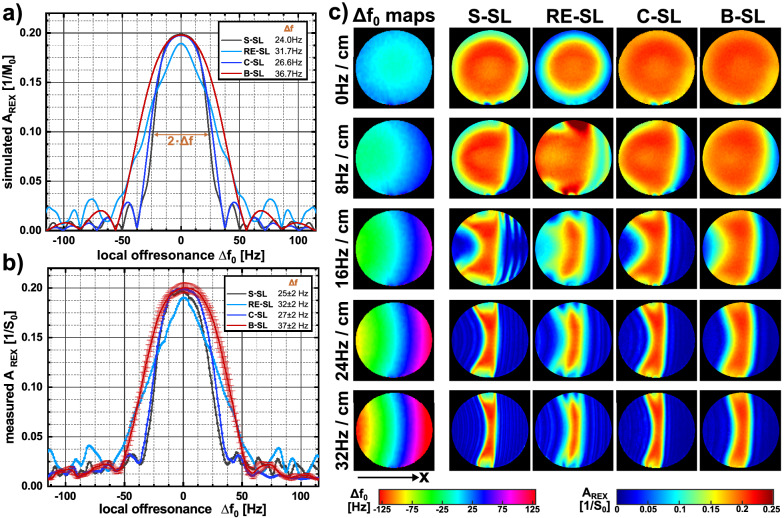
Performance of the REX modules in the presence of static B_0_ field inhomogeneities. Subfigure (**a**) shows the Bloch-simulated A_REX_ amplitudes, calculated for an offresonance range between − 1…1 ppm in 0.004 ppm steps (1 ppm ≙ 127.7 Hz at 3 T). Further simulation parameters were f_SL_ = f_stim_ = 50 Hz and $\Delta {\mathrm{B}}_{0}^{\mathrm{stim}}$ = 50 nT. The simulation was performed for optimal SL times 77.5 ms (S-SL), 70 ms (RE-SL), 80 ms (C-SL) and 80 ms (B-SL). The choice of these parameters is justified with respect to the results of the previous simulations (t_SL_ characteristics). The signal maximum is symmetrically distributed around zero and shows the widest maximum for the B-SL module. For offresonances > 50 Hz all modules indicate an oscillatory behavior. Experimental data were generated by applying an additional constant gradient (x direction), which projects a spatially-dependent field inhomogeneity within the tREX slice. The amplitude performance was determined by an ROI-based evaluation in which the measured amplitudes ${\mathrm{A}}_{\mathrm{REX}}(\vec{\mathrm{r}})$ were matched with the measured offresonances $\Delta {\mathrm{f}}_{0}(\vec{\mathrm{r}})$. The measurement results were averaged in 1 Hz intervals and compared with the simulation results. The signal amplitudes are plotted in (**b**) against the corresponding offresonance. For clarity, the error bars are only shown for B-SL. In (**c**), the field maps and the corresponding A_REX_ maps are shown for different static field inhomogeneities.

The results of the REX amplitude performance in presence of B_0_ inhomogeneities are shown in Fig. 6 and were compared with the corresponding results of the Bloch simulation. Both measurements and simulations show a decrease in A_REX_ with increasing $\Delta {\mathrm{f}}_{0}^{\mathrm{static}}$, confirming that static field inhomogeneities hamper the measurement of dynamic field oscillations. However, the four preparation modules show distinctly different sensitivities to B_0_ inhomogeneities. A halving of A_REX_ was observed in the simulation at the following $\Delta {\mathrm{f}}_{0}^{\mathrm{static}}$ values: ± 24.0 Hz for S-SL, ± 31.7 Hz for RE-SL, 26.6 Hz for C-SL, 36.7 Hz for B-SL (± 0.19 ppm, ± 0.25 ppm, ± 0.21 ppm, ± 0.29 ppm, respectively at 3 T). Thus, B-SL exhibits the highest and S-SL the lowest sensitivity (improvement ≈ 53%). In the phantom experiments we observed (25 ± 2) Hz, (32 ± 2) Hz, (27 ± 2) Hz and (37 ± 2) Hz, respectively. These results agree well in simulations and experiments. To minimize the influence of ${\mathrm{B}}_{1}^{+}$ deviation in the experimental validation, a ROI with small ${\mathrm{B}}_{1}^{+}$ variation (100.6 ± 3.2%) was evaluated.

### In vivo measurements

**Figure 7:**
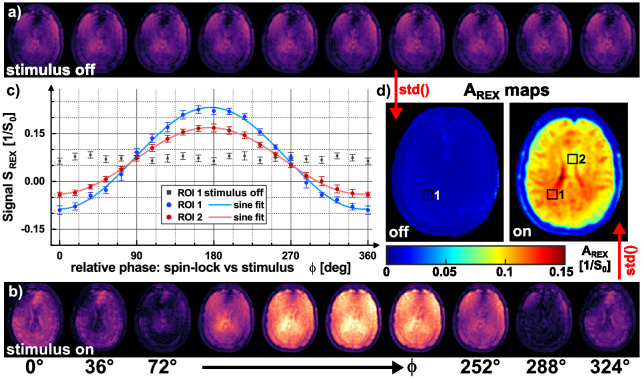
Proof-of-concept results of tREX measurements in vivo in human brain using B-SL. (**a**,**b**) REX weighted magnitude images (S_REX_) for 10 different relative phases between the spin-lock pulse and the magnetic field oscillation. REX weighted images with 20 relative phases ϕ were acquired in a Δz = 10 mm offcenter slice (f_SL_ = f_stim_ = 50 Hz, $\Delta {\mathrm{B}}_{0}^{\mathrm{stim}}$ = 50 nT) and control measurements were carried out (stimulus off). In (**c**), the signal intensities of the ROI based evaluation are plotted against the relative phase. As can be seen in the A_REX_ map in (**d**), the amplitude is higher in the first ROI than in the second. Both regions show, unlike the control measurement, a sinusoidal waveform with a 360° modulation. Structures of brain tissue and skull can be identified in both the REX weighted images and the A_REX_ map. The following parameters were used for spiral imaging: FOV = 240 × 240 mm^2^, matrix = 96 × 96, slice thickness 5 mm, T_rec_ = 1 s.

Figure 7 presents the proof-of-concept of the tREX method in vivo in brain tissue. The expected sinusoidal signal of rotary excitation was clearly observed in the REX weighted images (R^2^ > 0.99). No significant REX variation was detected in the control measurements (gradient stimulus off). In the calculated A_REX_ map, structures of brain tissue can be roughly distinguished. In two different ROIs, we observed ${\mathrm{A}}_{\mathrm{REX}}^{\mathrm{ROI},1}$ = 0.1192 ± 0.0037 and ${\mathrm{A}}_{\mathrm{REX}}^{\mathrm{ROI},2}$ = 0.0912 ± 0.0037. In the control experiments the reference amplitude was ${\mathrm{A}}_{\mathrm{REX}}^{\mathrm{ROI},1}$ = 0.00868 ± 0.00068 and ${\mathrm{A}}_{\mathrm{REX}}^{\mathrm{ROI},2}$ = 0.0076 ± 0.0025, respectively. The regions of skull bone and fat produced significantly reduced amplitudes.

**Figure 8:**
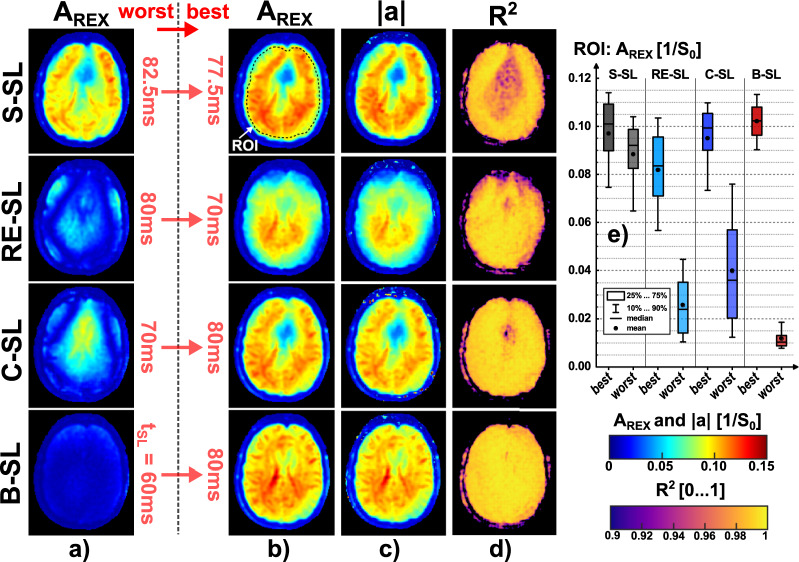
In vivo comparison of results for t_REX_ measurements with worst and best case choice of spin-lock pulse duration t_SL_. Except for the spin-lock duration, the identical sequence parameters as for Fig. 7 were used. (**a**) The A_REX_ results of the four modules with the respective worst choice of pulse duration. (**b**) Displays the corresponding best case measurements. (**c**,**d**) depict the REX fit results of |a| and the corresponding R^2^. The parameters A_REX_ and |a| show high agreement. A ROI-based evaluation of A_REX_ in brain tissue is presented in the box plot in (**e**). Especially for RE-SL, C-SL and B-SL, the high relevance of the pulse duration choice is evident. In case of an unsuitable choice, A_REX_ can be substantially reduced. For the S-SL module, as expected from the simulation results, there is no pronounced difference between best and worst settings. However, significant differences are also evident in the best case experiments for the compensated modules. Here, all modules except B-SL show reduced A_REX_ values near the cranial bone (RE-SL) or the frontal sinus (S-SL and C-SL). The overall best performance of B-SL is also evident in the R^2^ maps. The remaining fitted parameter maps and the results of B_0_ and ${B}_{1}^{+}$ mapping are presented in the Supplementary Material.

The impact of t_SL_ choice for B_0_ and ${\mathrm{B}}_{1}^{+}$ compensated modules is demonstrated by the A_REX_ maps in Fig. 8. While only minor differences were observed for S-SL, the remaining modules show clear differences between the best and worst case experiments. However, significant differences between the modules are also evident in the best case measurements. Local reductions in A_REX_ were observed near the frontal sinus (for S-SL, RE-SL and C-SL) and a significant reduction is noticeable for RE-SL near the cranial bone. This is also evident in the box plot in Fig. 8, where A_REX_ was examined throughout the whole brain tissue. B-SL shows the best overall performance and a clear delineation between best/worst case measurements. The amplitude parameter |a| fitted according to Eq. (1) yields highly comparable detection maps with approximately identical characteristics. On average over all maps, A_REX_ was 3.3% above |a|. The R^2^ values in brain tissue (best case) of the complex signal fit yielded values: 0.982 ± 0.012 for S-SL, 0.985 ± 0.014 for RE-SL, 0.9907 ± 0.0071 for C-SL, 0.9929 ± 0.0033 for B-SL. The remaining parameters of the fitted maps and the corresponding ΔB_0_ and ${\mathrm{B}}_{1}^{+}$ field maps are attached in the Supplementary Material (Supplementary Figs. 9–10). Supplementary Fig. 8 presents the REX weighted images of the best/worst case measurements for all modules.

**Figure 9:**
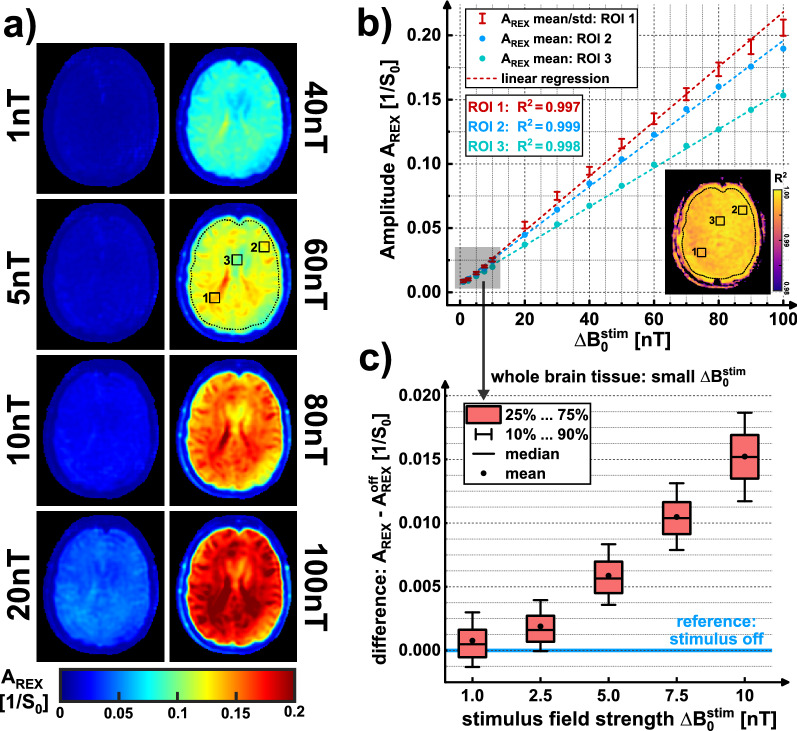
In vivo correlation measurements of the A_REX_ amplitude and the stimulus field strength $\Delta {\mathrm{B}}_{0}^{\mathrm{stim}}$. The A_REX_ values averaged within the marked ROIs for $\Delta {\mathrm{B}}_{0}^{\mathrm{stim}}$ between 1 and 100 nT (14 steps) from the maps in (**a**) are plotted against field strength in subfigure (**b**). Linear regression yielded R^2^ values > 0.99 for all three exemplary ROIs. The slope of the tREX calibration varies in different ROIs. The corresponding pixel-wise R^2^ map of the linear fit is also shown in (**b**). The averaged R^2^ in the whole brain tissue ROI (dashed line) was 0.9977 ± 0.0094. Due to the high agreement with the linear fit, tREX experiments enable in vivo calibration of REX amplitudes. This fact can be exploited in a fMRI experiment for quantifying neuronal magnetic field oscillations. The A_REX_ amplitude evaluated in the whole brain tissue, was examined more closely in the lower field strength range 1…10 nT in (**c**). The signal obtained for 1 nT cannot be significantly distinguished from the reference measurement (stimulus off) in the whole brain tissue. However, mean and median are both above the reference measurement amplitude (≈ 7.3%). Above 2.5 nT, the results provide a significant increase in A_REX_. A reason for the low separability at 1 nT could be the influence of respiration, as this increases the reference signal due to motion issues.

In the tREX calibration study, a clear linear correlation between the stimulus field strength $\Delta {\mathrm{B}}_{0}^{\mathrm{stim}}$ and the measured REX amplitude was observed under in vivo conditions. In Fig. 9, three exemplary ROIs were analyzed by means of linear regression and the agreement with a simple linear model yielded R^2^ > 0.99 in each case. Pixel-wise linear regression revealed R^2^ = 0.9977 ± 0.0094 in a whole brain tissue ROI. In addition, the whole brain tissue was evaluated specifically for small stimuli ($\Delta {\mathrm{B}}_{0}^{\mathrm{stim}}$ ≤ 10 nT). A slight rotary excitation effect could still be detected at 1 nT. Here, the average signal increase of A_REX_ compared to the reference measurement (stimulus off) was 7.3%. However, significant delineation to the reference signal in the brain tissue ROI was only possible at $\Delta {\mathrm{B}}_{0}^{\mathrm{stim}}$≥ 2.5 nT.

## Discussion

In the present work, a basic concept for the direct detection and quantification of neuronal activity by MRI has been advanced in three interdependent steps. The major concerns of the REX technique raised by Coletti^12^, namely the susceptibility to B_0_ and ${\mathrm{B}}_{1}^{+}$ inhomogeneities, were addressed by the implementation of established compensating spin-locking techniques^19–21^. Bloch simulations were used to progress the principle understanding of REX and its parameter dependency. The simulation results indicating significantly higher robustness of B_0_ and ${\mathrm{B}}_{1}^{+}$ compensated modules were confirmed in phantom experiments. For experimental validation purpose, the novel tREX approach was introduced. The transmission of rotary excitation using the built-in gradient system enables fast sequence development and validation with a high degree of precision and reproducibility without the need of external hardware. Based on this principle, initial validations of the REX technique were successfully performed in vivo and an approach for the quantification of biomagnetism can be proposed.

Experimental validation of the tREX waveforms could confirm that ultra-weak magnetic field oscillations can be generated with the standard built-in gradient system. While the timing of the phases is fully adequate for the purpose of the REX experiments, the amplitudes of the oscillations are limited, due to the precision of the hardware components. Ultimately, digital quantization of the electronic gradient control is the lower boundary. Minimum gradients of 0.1 nT/mm could only be just generated, whereby the detected waveforms were extensively noisy (R^2^ = 0.67). Thus, an oscillation of 1 nT within the isocenter near slice Δz = 10 mm is currently the limit of feasibility. Above this limit (1 … 20 nT/mm), reliable amplitude control and linearity could be demonstrated and the oscillations show the desired sinusoidal profile (R^2^ > 0.99). These technical conditions must be considered in tREX experiments especially if measurements are to be performed in more distant offcenter slices. In addition, tREX represents only a simple mimic of the in general spatially complex neuronal fields, because only z-components are generated and phase dispersion within a voxel is not produced.

The simulation and measurement results concerning REX based field detection show that an appropriate choice of SL pulse duration depending on the stimulus frequency is mandatory for the compensated RE-SL, C-SL, and B-SL modules. For this, a prediction rule (Eq. 4) was empirically developed. The accuracy of the prediction is reliable with a deviation of − 0.05%. Only for S-SL the prediction is less accurate (deviation of − 4.6%). Here, relaxation effects seem to systematically shift the position of the A_REX_ maxima. For the compensated modules, no significant influence of relaxation on the positions of local maxima could be found. Here, spin dynamic effects are crucial, since by reversing the SL direction or by applying refocusing pulses, the magnetization trajectory is abruptly bifurcated. Thus, constellations exist in which the final magnetization is projected either completely on the z-axis (maximum condition) or completely on the y′-axis (minimum condition). This phenomenon was illustrated in the Supplementary Material (Supplementary Fig. 2). The positions of the maxima conditions could be largely confirmed in the phantom experiments. The average deviation from the predicted positions was 5.5%. This can be explained mainly by a variation of the actual ${\mathrm{B}}_{1}^{+}$ field, whereby the SL amplitude is not exactly 50 Hz.

The present work addressed the question whether dynamic magnetic field oscillations can be measured with a simultaneous compensation of static field inhomogeneities. Bloch simulations verified that B-SL, for example, exhibits a significant increase in B_0_ robustness. A halving of the measured REX amplitude occurs from $\Delta {\mathrm{B}}_{0}^{\mathrm{static}}$= ± 0.29 ppm at 3 T. Thus, the REX method has functionality for typical B_0_ distributions in brain tissue after standard shimming. This was also evident in vivo, as B-SL still provided high A_REX_ sensitivity even in regions of increased B_0_ imperfections. The impact of ${\mathrm{B}}_{1}^{+}$ inhomogeneities was preventively compensated by using adiabatic excitation pulses as proposed in^22^. However, this only improves the initial conditions prior to the actual REX effect. A deviation in ${\mathrm{B}}_{1}^{+}$ still leads to a violation of the REX resonance condition (f_SL_ ≠ f_stim_). This cannot be fully compensated by any of the SL modules considered. Yet, in the simulations and measurements, B-SL indicated broader peaks in the t_SL_ characteristics, which effectively compensates for higher ${\mathrm{B}}_{1}^{+}$ deviations. This effect was also visible in the phantom experiment (Fig. 4). Despite an approximately optimal shim throughout the entire phantom (Δf_0_ = − 2.3 ± 3.5 Hz), a decrease in A_REX_ was observed at the edge of the phantom. In this region, deviations of the ${\mathrm{B}}_{1}^{+}$ field (≈ 90%, Supplementary Fig. 7) were present. In contrast to S-SL, B-SL generated approximately constant A_REX_ values. Another way to increase ${\mathrm{B}}_{1}^{+}$ robustness is the implementation of ramped SL pulses, which has been proposed in^18^. This may also achieve a broadening of the resonance effect for compensated REX modules. However, it became clear that even B-SL provides only the opportunity for REX detection at off-resonances below ≈ 50 Hz. This is currently a restriction that is particularly relevant for 3D field detection in the whole brain. For separate 2D scans, slice-selective higher order shimming is recommended. Another alternative is to adjust the carrier frequency of the spin-lock pulse to the local Larmor frequency of a specific ROI in the cortex.

The findings of simulation and phantom measurements were used to perform initial proof-of-concept experiments for oscillating magnetic field detection in vivo. Here, a spiral readout was used consistently, generating one REX-weighted image for each preparation. In contrast to the method proposed in^4^, not a true single-shot readout was used, but an interleaved acquisition with ramped flip angles. This step makes the readout less susceptible to off-resonance effects and still provides a quasi-single-shot in the context of the preparation. Thus, the sequence presented in this work can be applied for the detection of neuronal activity, where the relative phase of the stimulus is unknown and full k-space sampling must be carried out for each preparation. Another advantage of this technique is the reduced T_2_* effect due to the shorter individual readouts. This could also be beneficial for the robust detection of neuronal fields at high field strength, as the influence of the BOLD effect is effectively reduced by shorter spiral acquisition times. A disadvantage of our readout is the additional impact of T_1_ relaxation between the readouts and potential artifacts due to incorrect flip angles. Here, the use of Shinnar–Le-Roux optimized RF pulses and parallel imaging approaches could be explored for further improvement and acceleration^29–31^.

Although in vivo REX detection with the simple S-SL module was successful in principle, it was demonstrated that the use of B_0_ and ${\mathrm{B}}_{1}^{+}$ compensated modules provides significant improvement. In the detailed evaluation of the measured REX signals by the fit according to Eq. (1), significant increase in performance can be observed for C-SL and B-SL (Supplementary Fig. 8). In brain tissue these techniques yield homogeneous A_REX_ maps, low offsets of REX oscillation, and the signals are approximately 2π-periodic. Another finding was that RE-SL provided slightly better detection than C-SL in the region near the frontal sinus. This can be explained by the characteristics of the B_0_ susceptibility, where RE-SL clearly differs from the other modules and shows improved sensitivity at some high offresonance values. However, both in phantom and in vivo, RE-SL shows poor REX sensitivity at the edge of the object which is possibly linked to the ${B}_{1}^{+}$ distribution. Hence, in conclusion, we recommend to use at least the single refocused C-SL module or the double refocused B-SL module for in vivo REX experiments.

At present, a major challenge for REX detection lies in relatively high noise of the reference scans. In the reconstruction and evaluation of REX weighted images, the influence of respiration has not been corrected yet, which leads to increased ${\mathrm{A}}_{\mathrm{REX}}^{\mathrm{off}}$ and hampers separability at low $\Delta {\mathrm{B}}_{0}^{\mathrm{stim}}$. Therefore, as has been indicated by Truong et al.^4^, the true limit of sensitivity of the REX technique might be lower than 1 nT and can be further improved in vivo. This important point needs to be investigated in future studies, for example, using t-test statistics and repetitive REX experiments. Further investigations should be carried out whether other physiological processes such as blood flow impact REX detection and whether, e.g., triggered measurements or measurements during breath hold are beneficial. For motion correction, image registration techniques could be adapted from established BOLD fMRI routines^32,33^.

An important finding of our work is that tREX experiments establish a linear correlation (R^2^ > 0.99) between the artificial stimulus field strength and the measured REX amplitude in vivo. Thus, tREX offers high potential as a calibration technique for the amplitude quantification of biomagnetic fields. A reasonable approach for a REX fMRI protocol would be to measure two A_REX_ maps using tREX at different $\Delta {\mathrm{B}}_{0}^{\mathrm{stim}}$ and to calculate a tissue specific and spatially dependent REX calibration function. This can be utilized for the quantification of magnetic field oscillations in real measurements of neuronal activity by linking the measured REX amplitudes to the linear calibration function. It is essential to note that the tREX calibration depends on the selected offcenter slice and ultra-low stimulus field strengths (< 10 nT) cannot be generated throughout the whole volume of the brain due to the limited precision of the gradient system. For this reason, we propose for a robust determination of the calibration function to perform tREX measurements at stimulus field strengths well above this limit (e.g. 20 nT and 40 nT) and to exploit the linear correlation of the REX effect for the quantification of neuronal fields. However, a fixed limitation of the tREX method is that no calibrations can be performed in an isocenter slice. Thus, future fMRI studies should begin to trial tREX calibrations and subsequent REX detections in basic single-slice experiments near the isocenter.

In future studies, to achieve the next step for direct detection and quantification of neuronal fields, concepts for fMRI experiments based on a reliable method for modulating neural oscillations needs to be developed. One option for this is the state-dependent enhancement of alpha waves as suggested in^4^ or by triggering steady state visually evoked potentials (SSVEPs) with flickering stimuli^34,35^. Another promising application is the localization of the seizure onset zone in focal epilepsy^36^. While the expected stimulus frequencies in alpha activity and SSVEPs are below the 50 Hz oscillation used in this work, frequencies > 100 Hz can be expected in focal epilepsy. The stimulus frequency dependent prediction rule of A_REX_ maxima conditions found in this work can serve for optimization of the respective sequence parameters. As the relative phase with respect to the spin-lock is generally unknown, the REX amplitude must be determined by the standard deviation of random phase signals as discussed in^4,12^. A potential but technically complex alternative is the simultaneous recording of an EEG for retrospective evaluation and a subsequent detection by the REX fit presented in this work^37^. In this case, the R^2^ value of the REX fit could be used for an improved validation and higher statistical significance of the measured amplitudes. For these ambitious goals, the present work provides basic MRI concepts, specific optimization approaches and a pulse sequence that is already fully suitable for in vivo studies.

## Conclusion

The present work demonstrates a significant stabilization of spin-lock based detection of ultra-weak magnetic field oscillations. The findings concerning compensated REX modules provide a considerably more robust detection in the presence of field inhomogeneities and enable optimization specific to the stimulus frequency. Furthermore, the novel tREX validation technique allows sequences to be tested in phantom experiments as well as under controlled in vivo conditions. Hereby, the feasibility of magnetic field detection in the low nT range could already be demonstrated in brain tissue. Moreover, tREX could pave the way for quantifying amplitudes of neural field oscillations as robust calibration scans can be performed.

## Supplementary Information

Supplementary Information.

## Data Availability

The datasets used and/or analyzed during the current study are available from the corresponding author on reasonable request.

## Code availability

The original Pulseq MRI sequences for in vivo REX detection used in this work are provided in an open-access GitHub repository (https://github.com/maximiliangram/Pulseq_tREX).
