# Supplementary material for: Towards robust in vivo quantification of oscillating biomagnetic fields using Rotary Excitation based MRI
Source: Sci Rep. 2022 Sep 13;12:15375. doi: 10.1038/s41598-022-19275-5 (PMC9469076; doi:10.1038/s41598-022-19275-5)
Supplement: Supplementary file 1 — Supplementary Information. [file 41598_2022_19275_MOESM1_ESM.pdf]

# SUPPLEMENTARY MATERIAL

# Towards robust *in vivo* quantification of oscillating biomagnetic fields using Rotary Excitation based MRI


Maximilian Gram[1,2,†], Petra Albertova[1,2,†], Verena Schirmer[1], Martin Blaimer[3], Matthias Gamer[4], Martin J. Herrmann[5], Peter Nordbeck[2,‡], and Peter Michael Jakob[1,‡]

[1] *Experimental Physics 5, University of Würzburg, Würzburg, Germany*

[2] *Department of Internal Medicine I, University Hospital Würzburg, Würzburg, Germany*

[3] *Fraunhofer Institute for Integrated Circuits IIS, Würzburg, Germany*

[4] *Department of Psychology, University of Würzburg, Würzburg, Germany*

[5] *Department of Psychiatry, Psychosomatics, and Psychotherapy, Center for Mental Health, University Hospital of Würzburg, Würzburg, Germany*

[†] *these authors contributed equally to this work*

[‡] *these authors are joined supervisors*

**Address for correspondence:** Maximilian Gram, Experimental Physics 5, Faculty of Physics and Astronomy, University of Würzburg
Am Hubland, Würzburg, D-97074
maximilian.gram@physik.uni-wuerzburg.de.de
orcid.org/0000-0003-2184-3325


**List of contents:**


**S.1** Approaches and developments in spin-lock based magnetic field detection
**S.2** REX preparation modules
**S.3** Calculation of the spin-lock trajectories
**S.4** Simulation of $A_{REX}$ characteristics
**S.5** Validation of gradient waveforms for tREX experiments
**S.6** Extended measurement results


**Attached Supplementary Figures:**

The methods and results discussed in the following Supplementary Material are intended to provide a deeper understanding of the findings presented in the main manuscript. In particular, a brief summary of previously published approaches of spin-lock based magnetic field detection is given. The different REX preparation modules are discussed. The procedure for calculating magnetization trajectories using Bloch simulations is described. Additional simulations for investigation of the influence of parameters on $t_{SL}$ characteristics not discussed in the main manuscript are presented. Furthermore, supplementary results of phantom and *in vivo* experiments are presented for the validation of the tREX method.

# S.1 Approaches and developments in spin-lock based magnetic field detection

The initial approach (SIRS) for spin-lock based magnetic field detection was introduced in 2008 by Witzel et al [1]. The SIRS sequence starts with a 90° excitation pulse. The spin-lock pulse is subsequently applied resonantly and in phase to the transverse magnetization. After the SL pulse duration $t_{SL}$, another 90° pulse stores the magnetization in longitudinal direction. If there is no resonant external field in the sample volume during the SL pulse, the magnetization relaxes with $T_{1\rho}$ during $t_{SL}$. If, on the other hand, resonant interaction occurs, a secondary magnetic resonance effect arises and the magnetization tilts away from the SL axis (tilting angle $\theta$). The magnetization along the SL axis, which is stored in longitudinal direction before imaging, decreases beyond the $T_{1\rho}$ decay. The measured signal saturation compared to an experiment without resonant magnetic field interaction was estimated to be about 0.1% for realistic parameters using Bloch simulations [1]. Although the rotary saturation effect is small, ultra-weak magnetic oscillations up to 0.2nT could be detected with SIRS in phantom experiments [2].

In 2016 Jiang et al has found that the SIRS sequence in its original form has an adverse signal scaling behavior with respect to the tilting angle $\theta$ ($S_{SIRS} \propto 1 - cos(\theta) \approx \theta^2/2$, for small $\theta$) [3]. Therefore, Jiang proposed a positive contrast technique by directly measuring not the saturated magnetization component along the SL axis, but the excitation component perpendicular to it. In this context, a resonant neuronal magnetic field oscillation (stimulus frequency $f_{stim} = f_{SL}$) acts like a pseudo RF pulse, which leads to spin excitation in the rotating frame [4]. According to Redfield's nomenclature, we consequently referred to this effect as rotary excitation and abbreviate it as REX (Rotary EXcitation). The magnitude of $M_{REX}$ has a proportionality $sin(\theta) \approx \theta$ for small tilting angles, resulting in a significant signal increase ($S_{REX} \gg S_{SIRS}$) [3]. In addition, the final direction of $M_{REX}$ is sensitive to the relative phase $\phi$ between the SL pulse and the external field [5].

This modified approach shows increased sensitivity in simulations and phantom experiments compared to the SIRS technique [6, 7]. Extensive animal experiments in rats demonstrated the *in vivo* detection of artificially generated magnetic fields as low as 0.5nT at physiological noise conditions in brain tissue [7]. In 2019, Truong et al simplified this technique by omitting the second 90° flip back pulse of the SL preparation and examined imaging of alpha activity in combination with single-shot spiral acquisitions for the first time in human experiments [8]. By varying the relative phase $\phi$ between SL and the neuro-electro-magnetic oscillations (NEMO), a sinusoidal variation of $S_{REX}$ is detected. *In vivo*, $\phi$ is randomly varied and the standard deviation of the measured signal was evaluated for direct detection of neuronal activity. However, limitations of the method regarding susceptibility to $B_0$ and $B_1^+$ field inhomogeneities were identified in phantom measurements, in first results on human brain examinations and in statistical considerations [8, 9].

# S.2 REX preparation modules

The SL preparation modules originally developed for $T_{1\rho}$ quantification, which were used for REX preparation in this work, are compared in Supplementary Figure 1. The basic concepts of $B_1^+$ and $B_0$ compensated modules are assigned to the following original papers: rotary-echo spin-lock (RE-SL) [10], composite spin-lock (C-SL) [11], balanced spin-lock (B-SL) [12]. The concept of adiabatically prepared spin-locking was suggested by [13].

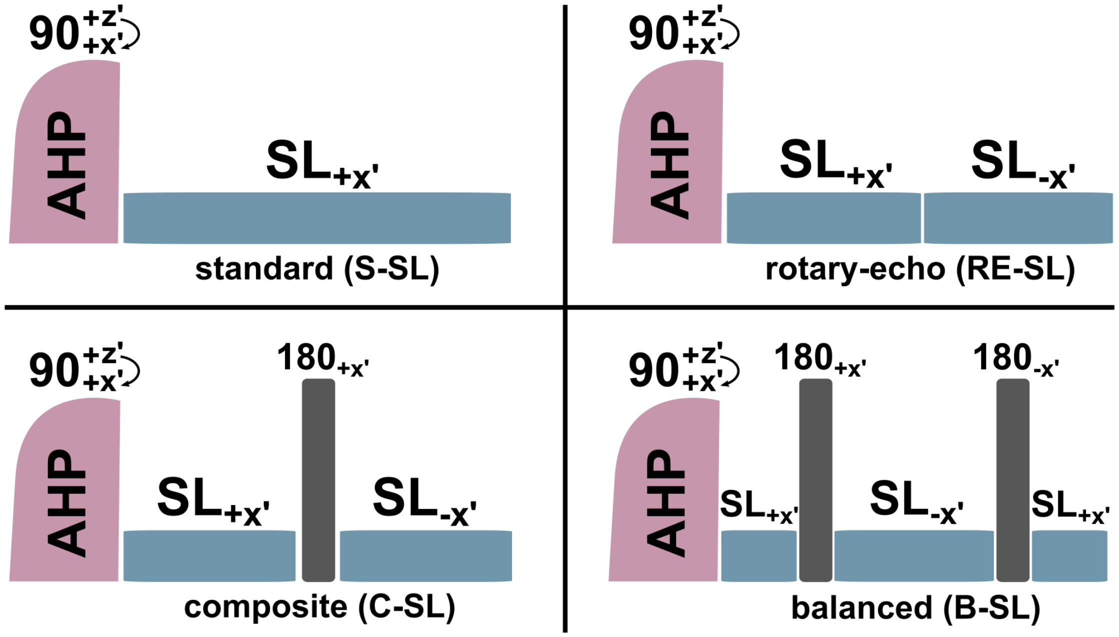


**Supplementary Figure 1)** Sequence diagrams of the four SL preparation modules. In the S-SL module, an adiabatic half passage (AHP) pulse is used to tilt the magnetization from the +z' direction to the +x' axis (rotating frame coordinates). Subsequently, a continuous wave spin-lock pulse is applied along the +x' axis. The RE-SL module is modified compared to S-SL, by dividing the SL pulse into two parts with antiparallel directions. This bifurcation ensures that the SL pulse as a whole does not lead to an effective rotation of the magnetization. Thus, an insensitivity to $B_1^+$ inhomogeneities can be achieved. In the C-SL module, an additional 180° pulse is inserted between the two spin-lock pulse blocks for additional $B_0$ compensation. The B-SL module contains a three-part SL pulse, where half of the SL duration is applied along the +x' direction and the other half along the –x' direction. Also an additional 180° pulse in the opposite -x' direction is inserted. Thus, all rotations are effectively canceled and the module is totally balanced.

## S.3 Calculation of the spin-lock trajectories

For the simulation of the parameter-dependent $A_{REX}$ sensitivity, the calculation of magnetization trajectories by an integration of the Bloch equations is required. The integration of the solution function starts with $\vec{M}'(t=0) = \vec{M}'_{init} = [M_0, 0, 0]^T$ at the beginning of the interaction between SL and stimulus. $M_0 = 1$ was set. The Bloch equations can be described as follows [6]:

$$\frac{d}{dt}\begin{bmatrix} M'_x \\ M'_y \\ M'_z \end{bmatrix} = \begin{bmatrix} -1/T_{1\rho} & \Delta\omega_0^{static} + \Delta\omega_0^{stim}(t) & 0 \\ -\Delta\omega_0^{static} - \Delta\omega_0^{stim}(t) & -1/T_{2\rho} & -\omega_{SL} \\ 0 & \omega_{SL} & -1/T_{2\rho} \end{bmatrix} \cdot \begin{bmatrix} M'_x \\ M'_y \\ M'_z \end{bmatrix} \tag{5}$$

Here $T_{1\rho}$ and $T_{2\rho}$ are the rotating frame relaxation times and $\omega_{SL} = 2\pi\, f_{SL} = \gamma B_{SL}$ is the SL amplitude. The parameter $\Delta\omega_0^{static} = 2\pi\, \Delta f_0$ was used for the consideration of static $B_0$ imperfections. The stimulus was assumed to be sinusoidal:

$$\Delta\omega_0^{stim}(t) = \gamma \cdot \Delta B_0^{stim} \cdot sin[2\pi f_{stim} \cdot t + \phi] \tag{6}$$

where $\gamma$ is the gyromagnetic ratio. The effect of 180° pulses for the modules C-SL and B-SL was simulated by matrix propagators [11, 12]. Since the duration of refocusing pulses is usually small compared to $t_{SL}$ and their amplitude is significantly higher than $f_{SL}$, these interactions with the stimulus were neglected. For the calculation of an amplitude $A_{REX}$, 10 simulations with linearly spaced

phases $\phi = 0 \ldots 2\pi$ were carried out. For each individual simulation, the solution function for the 3D magnetization trajectory was integrated until $t = t_{SL}$. Subsequently, $A_{REX}$ was calculated using the standard deviation of the 10 longitudinal magnetization values at the end of the SL interaction. The step-size in the Runge-Kutta method was $2.5\mu s$. For the relaxation times, typical values for human cortical gray matter were assumed ($T_{1\rho} = 78ms$, $T_{1\rho} = 117ms$, [14, 15]) unless otherwise specifically stated.

## S.4 Simulation of $A_{REX}$ characteristics

In the following section extended results of the Bloch simulation are presented. Supplementary Figure 2 is intended to provide a geometric understanding of the magnetization trajectory. Thereby it becomes clear for which reason minima and maxima are formed in the time course of the SL pulse (see Figure 5 and 8 in the main manuscript). The component of the magnetization which is imaged as REX weighted contrast is the z component. Consequently, maximum $A_{REX}$ values occur whenever the magnetization is stored in the xz-plane. However, if the magnetization is stored in the xy-plane, the $M_z$ component is zero and thus the signal disappears regardless of the relative phase. It is important to distinguish the disappearance of the signal at certain phase relations from the general pulse duration dependent minimum condition. The relative phase varies in the tREX experiments and can be random in real *in vivo* fMRI experiments. Thus, determining $A_{REX}$ as a standard deviation is not a problem, since a field can be detected in a series of measurements. However, if the measurement is performed in a minimum condition due to the choice of SL duration, no signal occurs for any relative phase and the measurement cannot indicate the presence of a magnetic field. Supplementary Figure 3 and 4 show that the relaxation times of the examined tissue as well as the field strengths of the oscillating stimulus to be measured have no significant influence on the position of the $A_{REX}$ maxima with respect to the SL duration. This promises to maximize $A_{REX}$ also *in vivo*, where the field strengths as well as the relaxation times vary locally, using the determined optimal parameter sets.

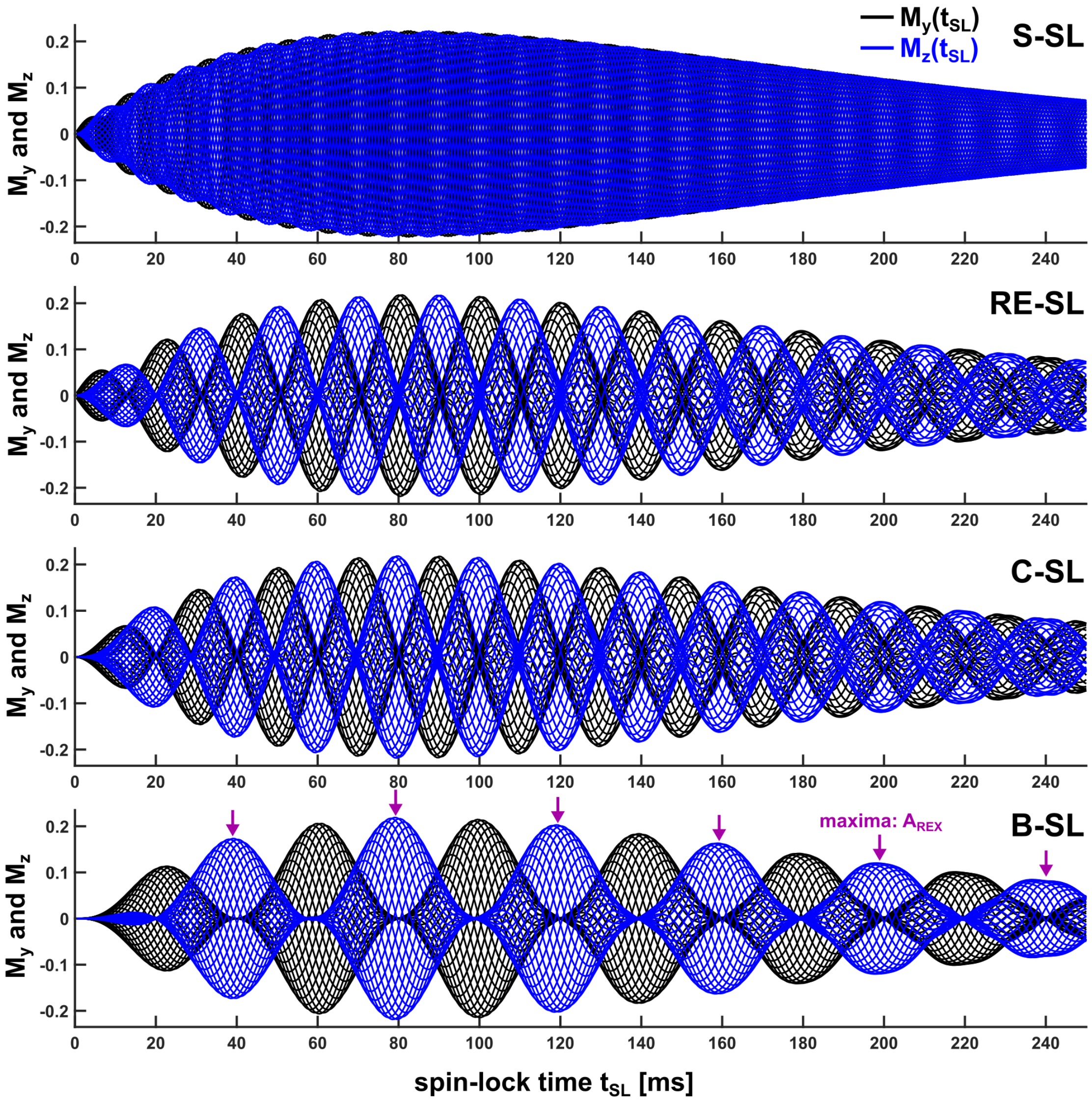


**Supplementary Figure 2)** Magnetization trajectories of the four preparation modules. The result of the Bloch simulation for the $M_y$ component (black) and the $M_z$ component (blue) as a function of the SL duration shows individual characteristics of the preparation module. The $M_z$ component is used for subsequent imaging. Consequently, $A_{REX}$ is maximized for SL durations for which the $M_z$ component is stored in z direction. For RE-SL, C-SL and B-SL conditions exist, which lead to zero $M_z$ storage. Here $A_{REX}$ is minimized.

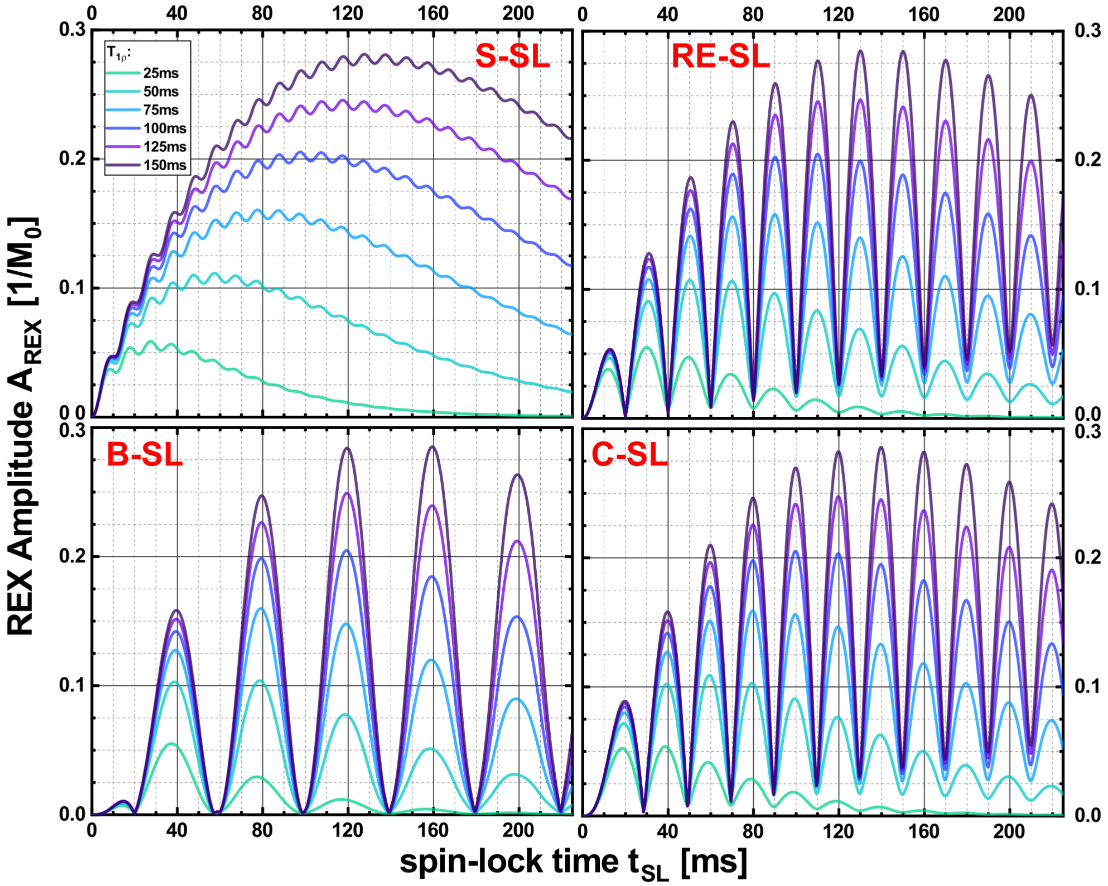


**Supplementary Figure 3)** Influence of the $T_{1\rho}$ relaxation time on the REX amplitude as a function of the SL pulse duration. Here, $T_{1\rho}$ was varied from $25 \ldots 150ms$ in $25ms$ steps and $T_{2\rho} = 1.5 \times T_{1\rho}$ was assumed. The REX amplitude shows maxima and minima for different SL pulse durations, as known from Supplementary Figure 2. $A_{REX}$ decreases with shorter $T_{1\rho}$ times, whereby the qualitative course and the position of local maxima is independent of the $T_{1\rho}$ time of the tissue under investigation. However, the relaxation times determine the position of the global maxima.

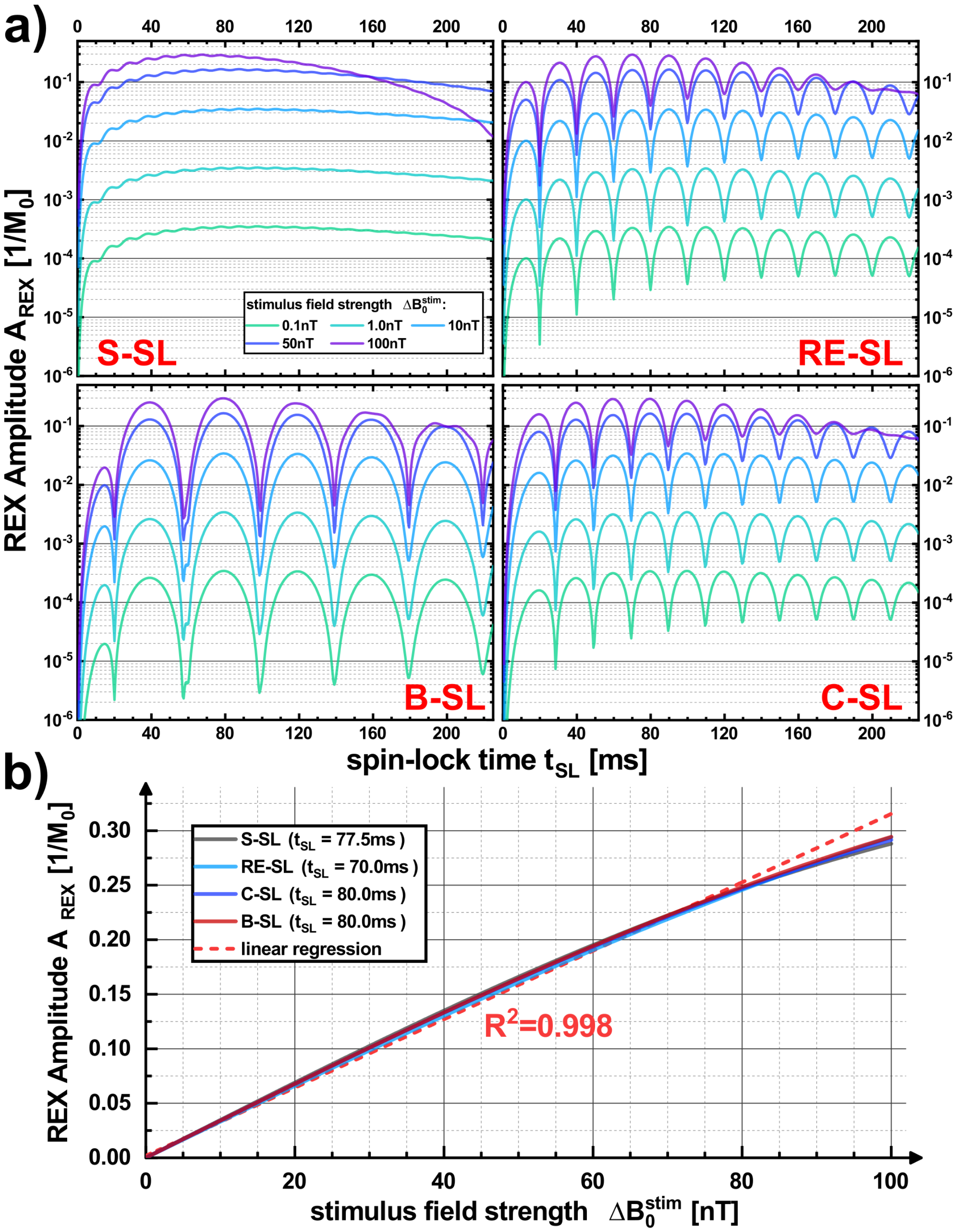


**Supplementary Figure 4)** Field strength dependence of the REX amplitude. **a)** Bloch simulated REX amplitude curves for different magnetic field strengths of the transmitted oscillating field. The field was assumed to be ideally sinusoidal with an amplitude between 0.1nT and 100nT. The qualitative course as well as the position of the maxima is essentially not dependent on the field strength for all four preparation modules. However, at high field strengths (100nT) a deformation can be seen. For high field strengths, a component perpendicular to the direction of the SL pulse is generated shortly after SL pulse onset. Consequently, $T_{2\rho}$ relaxation, which occurs in addition to $T_{1\rho}$ relaxation for components not aligned along the SL axis, has a noticeable effect for a longer duration at high field strengths. **b)** The course of the REX amplitude of the four modules at their respective optimal SL durations overlap, so that the individual plots are indistinguishable. The systematic deviation from the linear fit for high field strengths occurs due to saturation. As seen in subfigure a), the $T_{2\rho}$ relaxation plays an important role for high field strengths.

# S.5 Validation of gradient waveforms for tREX experiments

In order to validate the implemented tREX method, in which the magnetic field oscillation is projected onto the tissue under investigation, it must first be shown that the built-in gradient system can indeed produce waveforms in the lower nT range. For example, if rotary excitation with an oscillatory stimulus of 1nT was to be transmitted in a slice with an isocenter distance $\Delta z = 10$mm, the z-gradient amplitude has been set to 0.1nT/mm (according to Eq. 3 in the main manuscript). This corresponds to just 0.00022% of the nominal maximum value 45mT/m and thus represents a high technical challenge at the lower limit of the gradient system. Supplementary Figure 5 shows the measured gradient trajectories for different relative phases and amplitudes. The evaluation demonstrates that the built-in gradient system can be used for this purpose.

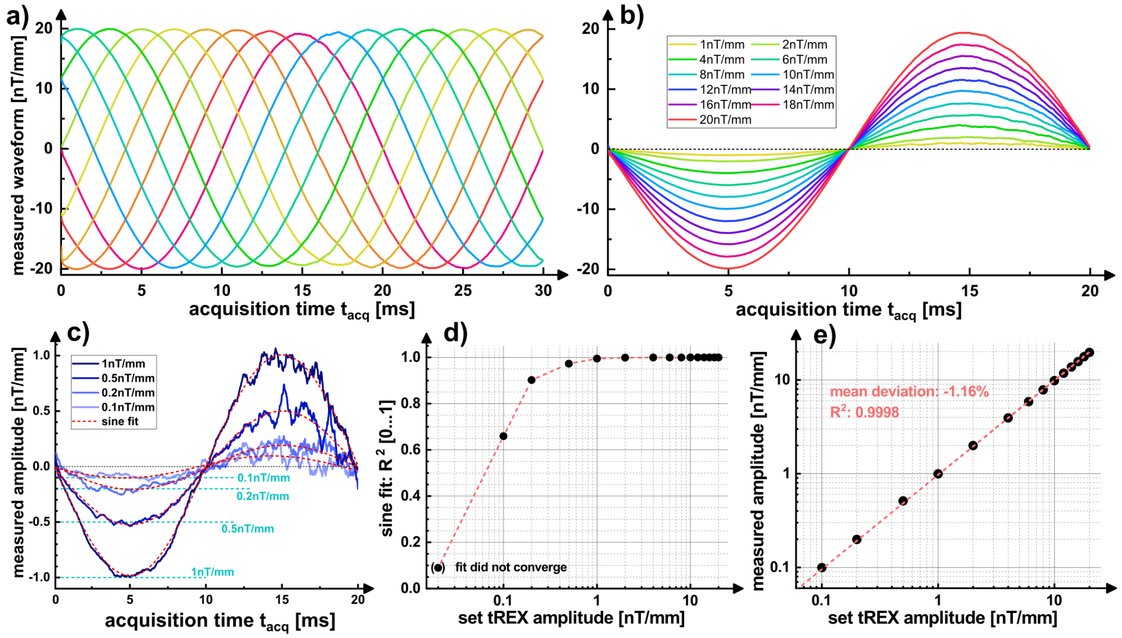


**Supplementary Figure 5)** Measurement of gradient trajectories for tREX validation at a stimulus frequency of 50Hz in the Agarose phantom in an offcenter slice $\Delta z = 25$mm (slice thickness 2mm). Subfigure **a)** shows the measured gradient trajectories for different relative phases $\phi$ $(0 \ldots 2\pi$, 10 steps) between SL pulse and the transmitted waveform with 100nT amplitude. The relative phase for the tREX measurements can be adjusted precisely. **b)** shows the measured gradient waveforms over one period for $\phi = 0$ and for different peak field strength values. The sinusoidal waveforms shown in **b)** and **c)** were fitted according to Eq. 1. The free parameters are the amplitude, the phase, the modulation and an offset. In **d)** the $R^2$ values of the fit for gradient oscillations between 0.1 and 20nT/mm (14 steps) are plotted. The fitted amplitudes illustrated in subfigure **e)** show high agreement with the set values over the examined range. Below 0.1nT/mm, however, no oscillation could be detected. In this range the limit of the accuracy of the gradient system is reached.

# S.6 Extended measurement results

Supplementary Figure 6 and 9 present the fitted parameter maps associated with the measurements in Figure 4, 7 and 8 of the main paper, respectively. Here all four modules are shown in comparison, whereas in the paper only selected results were shown. The pixel-wise fit according to Eq. 1 was performed in Matlab using implemented fminsearch() algorithms. According to Eq. 3, the amplitude $a(\vec{r})$ is proportional to the standard deviation, which serves as the measure of existing magnetic field oscillations under real conditions with non-adjustable phase. The parameter $b(\vec{r})$ indicates the offset. The signal oscillation with varying relative phase is not distributed around zero, but shows an offset

due to field inhomogeneities. The distribution of this parameter thus indicates the susceptibility of the preparation module to field inhomogeneities. Banding artifacts appear for all modules, but are clearly attenuated for C-SL and B-SL and the offset is reduced overall. The phase shift $\phi(\vec{r})$ shows structures in the phantom for the uncompensated S-SL module, while the phase is largely constant for the other modules within the sample. The modulation $m(\vec{r})$ is not equal to one if the frequency of the magnetic field oscillation is not exactly resonant to the spin-lock frequency (REX resonance condition). Supplementary Figures 7 and 10 show the corresponding $B_0$ and $B_1^+$ field maps in the phantom and in human brain. The REX weighted images corresponding to Figure 8 of the main paper are shown in Supplementary Figure 8.

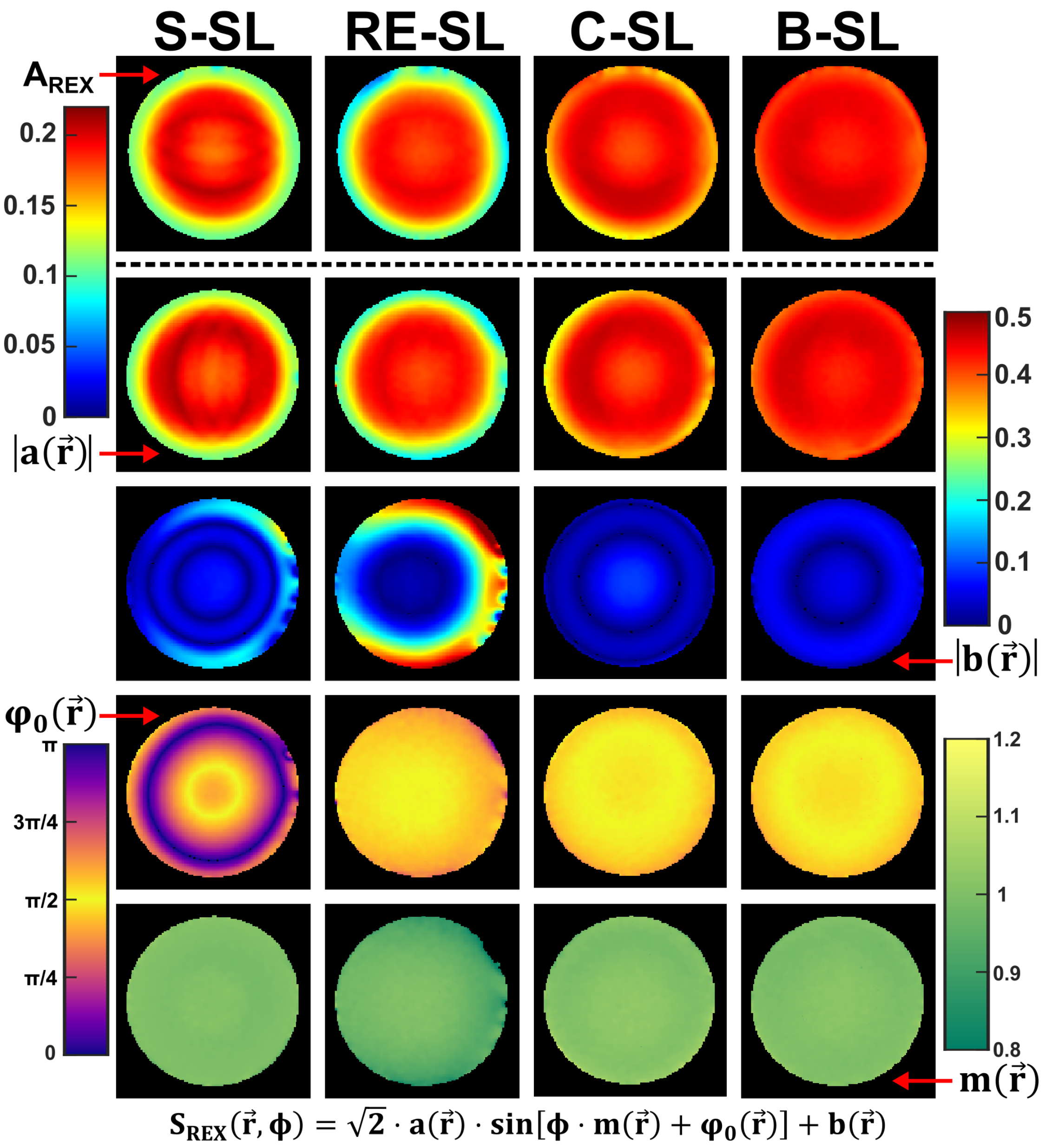


**Supplementary Figure 6)** Spatial distribution of $A_{REX}$ and the fit parameter values in the agarose phantom. 20 relative phases were measured at a stimulus frequency of 50Hz and 50nT field strength with optimal SL durations of the respective modules. The four fit parameters should be constant in the tREX slice under ideal conditions. However, $a(\vec{r})$ shows significant attenuation at the edge of the phantom for S-SL and RE-SL,due to $B_1^+$ inhomogeneities (Supplementary Figure 7). Furthermore, $b(\vec{r})$ shows increased values for S-SL and RE-SL and approximately zero magnitude for C-SL and B-SL. $\phi(\vec{r})$ shows variations within the tREX slice only at S-SL. The modulation parameter $m(\vec{r})$ is approximately one for all modules. A detailed evaluation can be found in the following Supplementary Table 1.

| | $A_{REX}$ | $\|a\|$ | $\|b\|$ | $\varphi_0$ | $m$ | $R^2$ |
|---|---|---|---|---|---|---|
| **S-SL** | $0.173 \pm 0.033$ | $0.169 \pm 0.032$ | $0.067 \pm 0.055$ | $1.498 \pm 0.906$ | $0.999 \pm 0.006$ | $0.999 \pm 0.001$ |
| **RE-SL** | $0.165 \pm 0.037$ | $0.160 \pm 0.033$ | $0.171 \pm 0.139$ | $1.726 \pm 0.123$ | $0.960 \pm 0.038$ | $0.998 \pm 0.003$ |
| **C-SL** | $0.193 \pm 0.017$ | $0.183 \pm 0.017$ | $0.030 \pm 0.023$ | $1.511 \pm 0.092$ | $1.009 \pm 0.012$ | $0.999 \pm 0.002$ |
| **B-SL** | $0.199 \pm 0.009$ | $0.190 \pm 0.009$ | $0.040 \pm 0.018$ | $1.526 \pm 0.091$ | $1.002 \pm 0.009$ | $0.999 \pm 0.001$ |

**Supplementary Table 1)** Detailed evaluation of $A_{REX}$, the fit parameters and $R^2$ maps corresponding to Supplementary Figure 6. The data are the mean and standard deviation values of the whole tREX slice. On average over all maps, $A_{REX}$ was 3.98% above $|a|$.

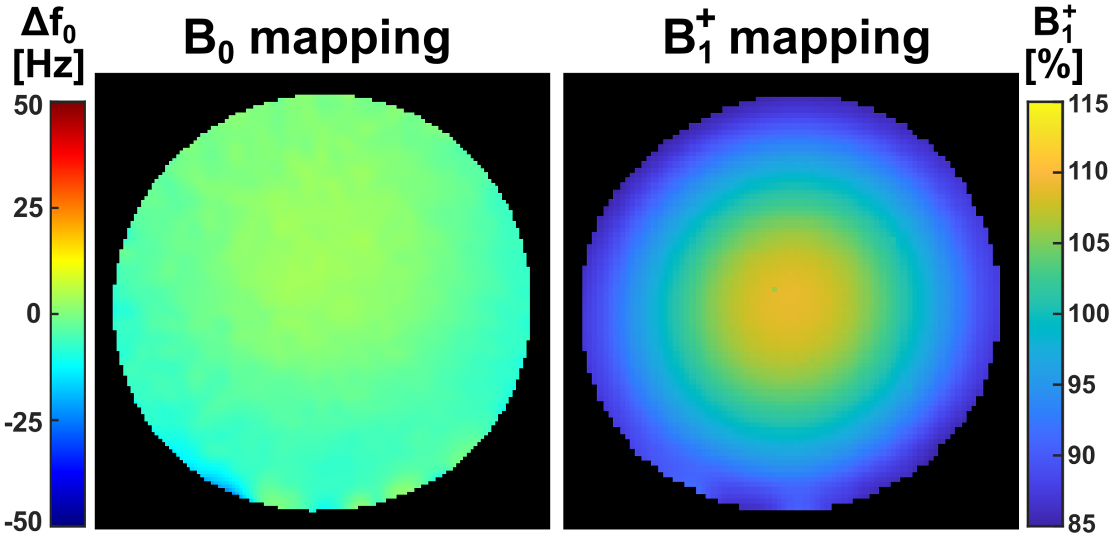


**Supplementary Figure 7)** Results of $B_0$ and $B_1^+$ mapping in the tREX slice in the phantom experiment. As can be seen in the left offresonance map, the field within the phantom shows a high homogeneity with a mean frequency variation of only $\Delta f_0 = -2.3 \pm 3.5$Hz. This indicates that the 2nd order shimming routine has a good performance in the experimental setup. The $B_1^+$ map in the phantom shows that higher SL amplitudes ($\approx 110 \ldots 115\%$) are present in the central, inner area of the phantom and lower SL amplitudes ($\approx 85 \ldots 90\%$) near the phantom edge. This leads to the REX resonance condition not being met throughout the whole tREX slice.

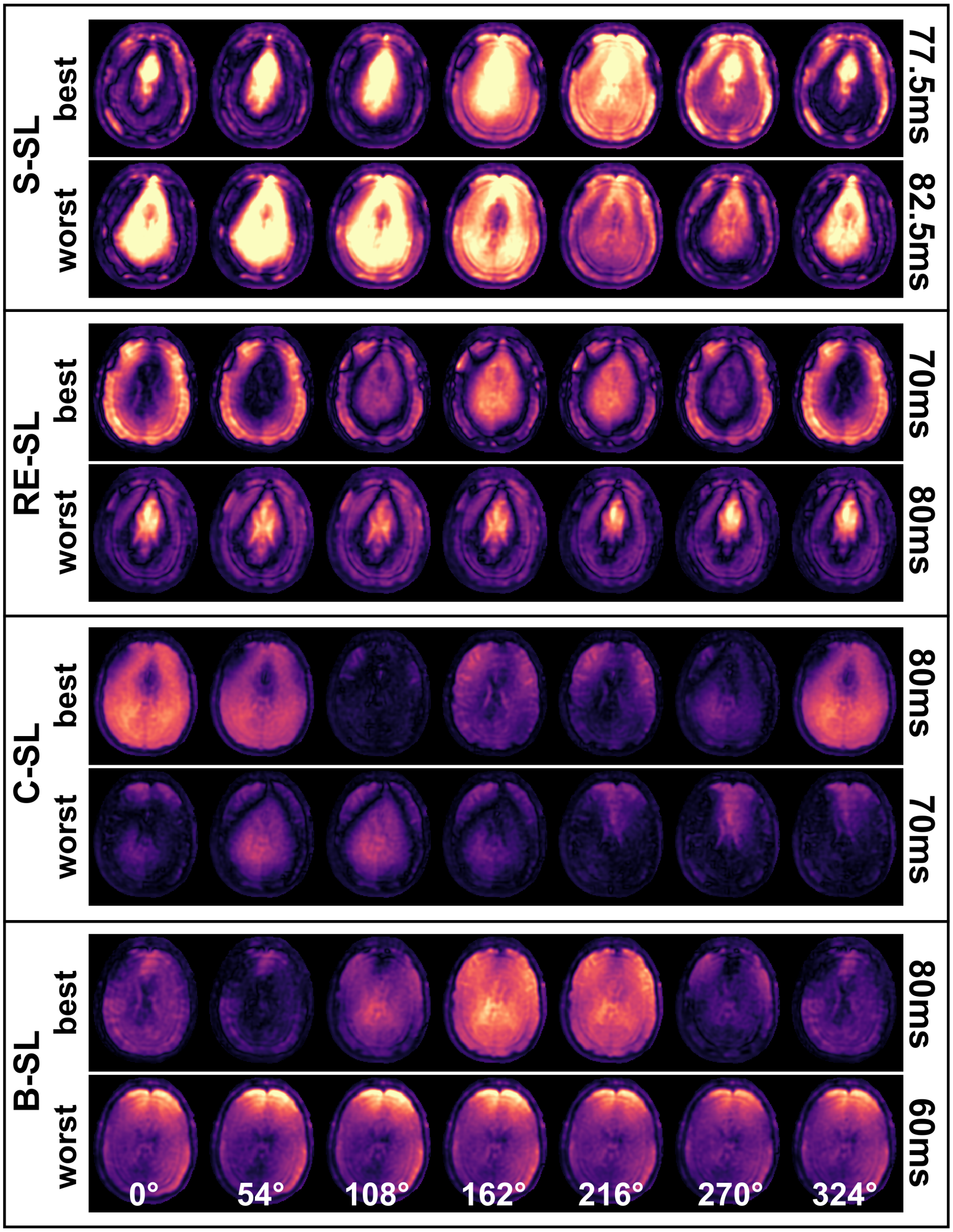


**Supplementary Figure 8)** Illustration of measured REX weighted magnitude images for best and worst case choice of SL pulse durations. The figure exemplary shows 7 of 20 relative phases used for $A_{REX}$ calculation.

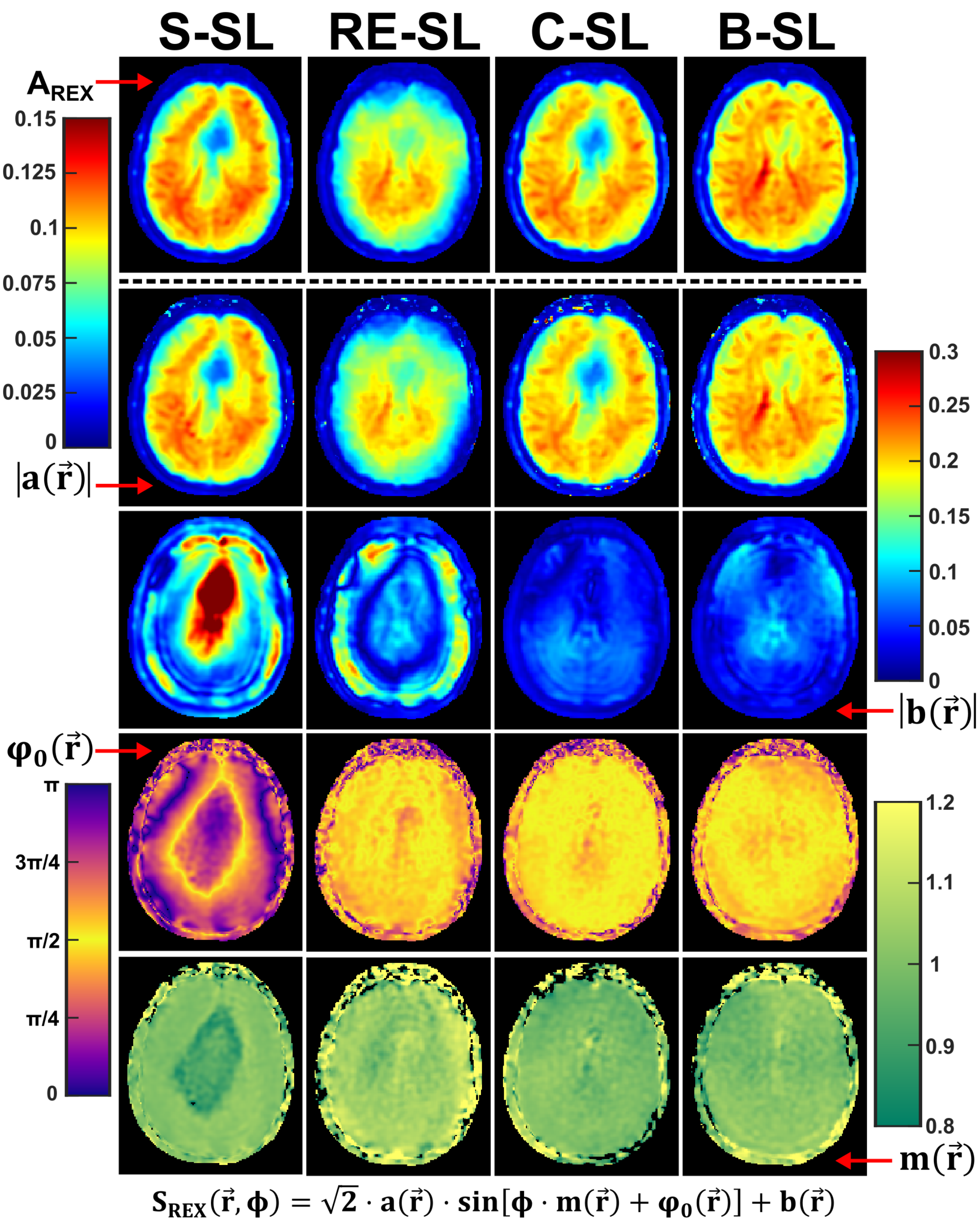


**Supplementary Figure 9)** Spatial distribution of $A_{REX}$ and the fit parameter values in the *in vivo* measurements. 20 relative phases were measured at a stimulus frequency of 50Hz and 50nT field strength with optimal SL durations of the respective modules. The fitted $a(\vec{r})$ maps are in good agreement with the calculated $A_{REX}$ maps in Figure 8 in the main paper calculated by standard deviations. Furthermore, $b(\vec{r})$ shows increased values for S-SL and RE-SL and significantly reduced magnitude for C-SL and B-SL. $\phi(\vec{r})$ shows significant variations within the tREX slice for S-SL and variations in the skull bone for all modules. The modulation parameter $m(\vec{r})$ is approximately one for C-SL and B-SL in brain tissue and exhibits clear inhomogeneities for S-SL and RE-SL. A detailed evaluation can be found in the following Supplementary Table 2 for brain tissue.

| | $A_{REX}$ | $\|a\|$ | $\|b\|$ | $\varphi_0$ | $m$ | $R^2$ |
|---|---|---|---|---|---|---|
| **S-SL** | $0.097 \pm 0.017$ | $0.095 \pm 0.017$ | $0.144 \pm 0.086$ | $1.454 \pm 0.794$ | $0.982 \pm 0.051$ | $0.982 \pm 0.012$ |
| **RE-SL** | $0.082 \pm 0.018$ | $0.078 \pm 0.017$ | $0.086 \pm 0.047$ | $1.473 \pm 0.129$ | $1.051 \pm 0.035$ | $0.985 \pm 0.014$ |
| **C-SL** | $0.095 \pm 0.016$ | $0.093 \pm 0.015$ | $0.052 \pm 0.019$ | $1.594 \pm 0.088$ | $0.995 \pm 0.026$ | $0.991 \pm 0.007$ |
| **B-SL** | $0.102 \pm 0.009$ | $0.099 \pm 0.009$ | $0.058 \pm 0.022$ | $1.540 \pm 0.122$ | $1.009 \pm 0.027$ | $0.993 \pm 0.003$ |

**Supplementary Table 2)** Detailed evaluation of $A_{REX}$, the fit parameters and $R^2$ maps corresponding to Supplementary Figure 9. The data are the mean and standard deviation values of the brain tissue ROI used in the main manuscript. On average over all maps, $A_{REX}$ was 3.33% above $|a|$.

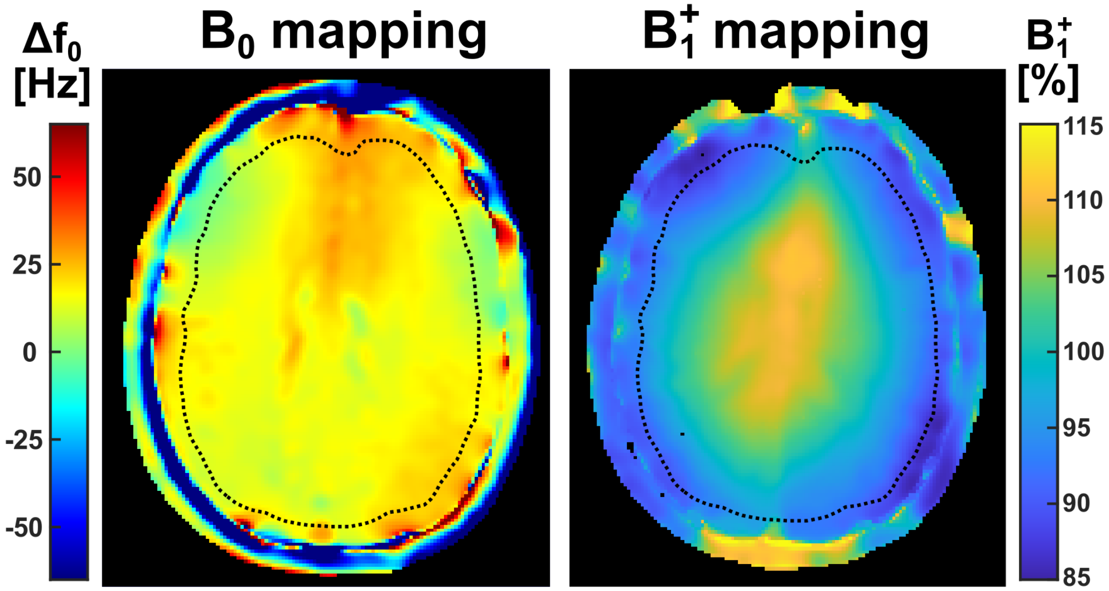


**Supplementary Figure 10)** Results of $B_0$ and $B_1^+$ mapping in the tREX slice in the *in vivo* experiment. The dashed line marks the brain tissue ROI, which was used in the main manuscript. $B_0$ field inhomogeneities are visible, especially near the frontal sinus, compared to the rest of the brain tissue. The field inhomogeneities outside the brain tissue, in the vicinity of the skull bone, are of no significance for the REX measurements, since this area is not of interest in application-related measurements. The average frequency offset $\Delta f_0$ in brain tissue was $15.9 \pm 4.8$Hz. The $B_1^+$ map shows a field distribution similar to the phantom experiments. The values range from $\approx 85 \ldots 115\%$ in the measured FOV and the mean and standard deviation in the brain tissue ROI is $99.9 \pm 6.2\%$.

## Supplementary references


**[1]** Witzel T, Lin FH, Rosen BR, Wald LL. *Stimulus-induced Rotary Saturation (SIRS): a potential method for the detection of neuronal currents with MRI.* Neuroimage. 2008 Oct 1;42(4):1357-65. doi.org/10.1016/j.neuroimage.2008.05.010

**[2]** Nagahara S, Ueno M, Kobayashi T. *Spin-Lock Imaging for Direct Detection of Oscillating Magnetic Fields with MRI: Simulations and Phantom Studies.* Advanced Biomedical Engineering. 2013;2:63-71. doi.org/10.14326/abe.2.63

**[3]** Jiang X, Sheng J, Li H, Chai Y, Zhou X, Wu B, Guo X, Gao JH. *Detection of subnanotesla oscillatory magnetic fields using MRI.* Magn Reson Med. 2016 Feb;75(2):519-26. doi.org/10.1002/mrm.25553

**[4]** Ito Y, Ueno M, Kobayashi T. *Neural magnetic field dependent fMRI toward direct functional connectivity measurements: A phantom study.* Sci Rep. 2020 Mar 25;10(1):5463. doi.org/10.1038/s41598-

020-62277-4

[**5**] Chai Y, Bi G, Wang L, Xu F, Wu R, Zhou X, Qiu B, Lei H, Zhang Y, Gao JH. *Direct detection of optogenetically evoked oscillatory neuronal electrical activity in rats using SLOE sequence.* Neuroimage. 2016 Jan 15;125:533-543. doi.org/10.1016/j.neuroimage.2015.10.058

[**6**] Ueda H, Seki H, Ito Y, Oida T, Taniguchi Y, Kobayashi T. *Dynamics of magnetization under stimulus-induced rotary saturation sequence.* J Magn Reson. 2018 Oct;295:38-44. doi.org/10.1016/j.jmr.2018.07.004

[**7**] Sheng J, Liu Y, Chai Y, Tang W, Wu B, Gao JH. *A comprehensive study of sensitivity in measuring oscillatory magnetic fields using rotary saturation pulse sequences.* Magn Reson Imaging. 2016 Apr;34(3):326-33. doi.org/10.1016/j.mri.2015.11.007

[**8**] Truong TK, Roberts KC, Woldorff MG, Song AW. *Toward direct MRI of neuro-electro-magnetic oscillations in the human brain.* Magn Reson Med. 2019 Jun;81(6):3462-3475. doi.org/10.1002/mrm.27654

[**9**] Coletti C, Domsch S, Vos F, Weingärtner S. *Functional MRI of neuro-electro-magnetic oscillations: Statistical processing in the presence of system imperfections.* 2020 IEEE-EMBS Conference on Biomedical Engineering and Sciences (IECBES). 2021;172-177. doi.org/10.1109/IECBES48179.2021.9398751

[**10**] Charagundla SR, Borthakur A, Leigh JS, Reddy R. *Artifacts in T(1rho)-weighted imaging: correction with a self-compensating spin-locking pulse.* J Magn Reson. 2003 May;162(1):113-21. doi.org/10.1016/s1090-7807(02)00197-0

[**11**] Witschey WR 2nd, Borthakur A, Elliott MA, Mellon E, Niyogi S, Wallman DJ, Wang C, Reddy R. *Artifacts in T1rho-weighted imaging: compensation for B(1) and B(0) field imperfections.* J Magn Reson. 2007 May;186(1):75-85. doi.org/10.1016/j.jmr.2007.01.015

[**12**] Gram M, Seethaler M, Gensler D, Oberberger J, Jakob PM, Nordbeck P. *Balanced spin-lock preparation for B1 -insensitive and B0 -insensitive quantification of the rotating frame relaxation time T1ρ.* Magn Reson Med. 2021 May;85(5):2771-2780. doi.org/10.1002/mrm.28585

[**13**] Schuenke P, Koehler C, Korzowski A, Windschuh J, Bachert P, Ladd ME, Mundiyanapurath S, Paech D, Bickelhaupt S, Bonekamp D, Schlemmer HP, Radbruch A, Zaiss M. *Adiabatically prepared spin-lock approach for T1ρ-based dynamic glucose enhanced MRI at ultrahigh fields.* Magn Reson Med. 2017 Jul;78(1):215-225. doi.org/10.1002/mrm.26370

[**14**] Watts R, Andrews T, Hipko S, Gonyea JV, Filippi CG. *In vivo whole-brain T1-rho mapping across adulthood: normative values and age dependence.* J Magn Reson Imaging. 2014 Aug;40(2):376-82. doi.org/10.1002/jmri.24358

[**15**] Wheaton AJ, Borthakur A, Corbo MT, Moonis G, Melhem E, Reddy R. *T2rho-weighted contrast in MR images of the human brain.* Magn Reson Med. 2004 Dec;52(6):1223-7. doi.org/10.1002/mrm.20284
